# Diversity of Pleosporalean Fungi Isolated from Rice (*Oryza sativa* L.) in Northern Thailand and Descriptions of Five New Species

**DOI:** 10.3390/jof10110763

**Published:** 2024-11-02

**Authors:** Sahar Absalan, Alireza Armand, Ruvishika S. Jayawardena, Eric H. C. McKenzie, Kevin D. Hyde, Saisamorn Lumyong

**Affiliations:** 1 Department of Biology, Faculty of Science, Chiang Mai University, Chiang Mai 50200, Thailand; saharabsalan6@gmail.com; 2 Center of Excellence in Fungal Research, Mae Fah Luang University, Chiang Rai 57100, Thailand; armandalireza.72@gmail.com (A.A.); ruvishika.jay@mfu.ac.th (R.S.J.); 3 School of Science, Mae Fah Luang University, Chiang Rai 57100, Thailand; 4 Landcare Research-Manaaki Whenua, Private Bag 92170, Auckland 1072, New Zealand; mckenziee@landcareresearch.co.nz; 5 Academy of Science, The Royal Society of Thailand, Bangkok 10300, Thailand; 6 Center of Excellence in Microbial Diversity and Sustainable Utilization, Faculty of Science, Chiang Mai University, Chiang Mai 50200, Thailand

**Keywords:** multi-gene analyses, paddy fields, Pleosporales, *Pyrenochaetopsis oryzicola*, taxonomy

## Abstract

Pleosporales represents the largest order within the class Dothideomycetes (Fungi), comprising phytopathogenic, saprobic, and endophytic taxa with a widespread presence in terrestrial and aquatic environments. Rice (*Oryza sativa*) is a primary economic crop in numerous tropical countries, particularly in Thailand. Studying fungal species associated with rice holds the potential to enhance our understanding of fungal diversity, lifestyles, and biology of rice, offering valuable insights for future research aimed at disease management and yield improvement. Thirty-nine pleosporalean isolates were obtained from various parts of rice plants collected across diverse regions in Chiang Rai Province, Thailand. Species identification involved a combination of morphology and molecular phylogeny, utilizing multi-locus sequence analyses of the ITS, LSU, SSU, *gapdh*, *rpb*2, *tef*1, and *tub*2 genes. The isolates were identified in 18 taxa distributed across five families and ten genera, including five new species (*Bipolaris chiangraiensis*, *Ophiosphaerella oryzae*, *Paraphaeosphaeria oryzae*, *Pyrenochaetopsis oryzicola*, and *Setophoma oryzicola*). Additionally, six new host records and two new geographical records are documented. Photoplates, detailed morphological descriptions, and phylogenetic trees are provided to elucidate the placement of both known and novel taxa.

## 1. Introduction

Rice (*Oryza sativa*, Poaceae) is grown in more than 100 countries, including the Middle East, the West Indies, and Latin America, with 90% of the total global production originating from Asian countries [1]. More than half of the world’s population consumes rice as a staple food, providing more than 20% of the calorie consumption globally because of its many nutrients, which contain fiber, vitamin B, proteins, and lipids [2].

In Thailand, rice is the most significant crop and is grown extensively across the country, comprising approximately half of the total cultivated area [3]. In the history of Thailand, rice is believed to have been originally cultivated as early as 3500 BC. More than 60% of the Thai population consists of farmers, with rice serving as the primary crop for the majority [4]. In 2022, Thailand exported approximately 7.7 million metric tons of rice [5], reinforcing its position as one of the world’s leading and most significant rice producers. However, production of rice in Thailand is affected by various factors.

Plants are exposed to a wide range of biotic and abiotic factors [6]. Among the biotic factors, are the microbial communities or microorganisms associated with plants. They are known as the plant microbiome or microbiota, with fungi playing essential roles, which can impact a host plant positively or negatively [7,8]. Fungi are among the most important pathogenic microorganisms in plants, especially for crop plants in agriculture; however, they are also used as biocontrol agents to prevent and manage plant diseases [9]. Rice is threatened by many pathogenic fungi leading to significant yield losses worldwide, and some of the taxa also release hazardous toxins which may be consumed [10,11]. Several fungi with different nutritional modes also exist in rice, providing many benefits to the plant [12,13,14].

The fungal order, Pleosporales was established by Luttrell [15] and legitimately proposed by Barr [16], primarily centered on *Pleosporaceae*, with the type species *Pleospora herbarum* [17]. It is the largest and most diverse order in Dothideomycetes (Ascomycota), encompassing approximately a quarter of all dothideomycetous species [18]. Pleosporalean species are distributed globally as lichenized fungi, pathogens, saprobes, endophytes, and parasites or hyperparasites on fungi and insects [19,20]. Pleosporales was recently listed with 91 families and more than 500 genera [21,22]. The sexual morphs are characterized by perithecioid ascomata, generally with a papillate apex, bitunicate, fissitunicate asci, septate ascospores of various colors and shapes, with or without a sheath [21]. Asexual morphs are mostly coelomycetous; however, sometimes they can be hyphomycetous [18,23,24,25]. This order comprises several important genera possessing both saprobic and pathogenic lifestyles on monocot plants worldwide, such as *Alternaria*, *Bipolaris*, *Curvularia*, and *Phaeosphaeria* [18,26,27,28,29,30].

Although many studies have reported fungi associated with rice worldwide, the fungal diversity on rice in Thailand is poorly understood. Hence, more investigations are required to discover and record the fungal species diversity in this strategic crop in Thailand. In this study, we focused on genera belonging to Pleosporales isolated from rice in northern Thailand.

## 2. Materials and Methods

### 2.1. Sample Collection and Specimen Examination

Rice samples (different tissue parts) of commonly cultivated varieties were collected from different locations in Chiang Rai Province, Thailand, from November 2021 to November 2022. Rice collections consisted of healthy tissues for isolating endophytes, dead plant material for obtaining saprobes, and the leaves with lesions in order to isolate fungi associated with symptoms. Details such as the collection date, location, and where possible, cultivar names were obtained and noted on each collecting bag and the samples were then transferred to the laboratory. The specimens underwent external observation using a Motic SMZ 168 stereomicroscope (Motic, Xiamen, China), and micro-morphological characteristics were studied using a compound microscope equipped with a digital camera (Nikon Eclipse 80i, Nikon, Minato City, Japan). Measurements were acquired using the Tarosoft (R) Image Frame Work program, and subsequent adjustments were made using Adobe Photoshop v. 21.1.3 (Adobe, San Jose, CA, USA).

### 2.2. Isolation

Fungi were isolated by using both direct and tissue isolation methods [31]. To purify the obtained strains, single spore and hyphal tip isolation techniques were employed, following the protocols outlined by Senanayake et al. [31]. Morphological characteristics were examined directly from the host or after a two-week incubation of obtained cultures. Dried herbarium specimens were archived in the Mae Fah Luang University Herbarium (Herb. MFLU), located in Chiang Rai, Thailand. Living cultures on potato dextrose agar (PDA) were deposited in the Culture Collection of Mae Fah Luang University (MFLUCC). Index Fungorum [32] and Faces of Fungi [33] numbers were obtained for novel taxa.

### 2.3. DNA Extraction, PCR Amplification and Sequencing

Genomic DNA was extracted from 200 mg mycelia scraped from two-week-old cultures using the OMEGA E.Z.N.A.^®^ Forensic DNA Kit (Omega Bio-tek, Norcross, GA, USA) following the manufacturer’s protocols. PCR amplification reactions were conducted to amplify partial gene regions of internal transcribed spacers (ITS), 28S ribosomal RNA (LSU), 18S ribosomal RNA (SSU), RNA polymerase II second largest subunit (*rpb*2), β-tubulin (*tub*2), glyceraldehyde-3-phosphate dehydrogenase (*gapdh*), and translation elongation factor 1–alpha (*tef*1) using appropriate primers (Table 1). The PCR mixture comprised 12.5 μL of 2 × Power Taq PCR MasterMix (a premix and ready-to-use solution containing Taq DNA Polymerase, dNTPs, and optimized buffer), 9.5 μL of deionized water, 1 μL of each primer (10 pM), and 1 μL of genomic DNA. Positive amplicons were assessed using agarose gel electrophoresis and visualized on a cybergreen-stained agarose gel under UV light using a molecular imaging system. Positive PCR products were forwarded to SolGent Co., Daejeon, Republic of Korea, for purification and sequencing, employing the same primers.

### 2.4. Sequence Alignment

Individual loci sequences were aligned using MAFFT v. 7.036 (http://mafft.cbrc.jp/alignment/server/large.html, accessed on 21 February 2024) [40] with default settings. Before phylogenetic analyses, the alignments underwent manual editing with BioEdit v. 7.0.5.2 [41]. Newly generated nucleotide sequences were submitted to GenBank, and corresponding accession numbers were included in the appropriate entries.

### 2.5. Phylogenetic Analyses

The genus of all isolates was confirmed using the BLASTn tool (Basic Local Alignment Search Tool; https://blast.ncbi.nlm.nih.gov/Blast.cgi, accessed on 5 October 2023) at the National Center for Biotechnology Information (NCBI), and related sequence data were subsequently obtained from GenBank and recently published papers [42,43,44,45]. Maximum likelihood (ML) analysis was carried out using RAxML-HPC2 on XSEDE v.8.2.8 [46,47] in the CIPRES Science Gateway platform [48] with the GTRGAMMA evolution model and bootstrap supports with 1000 replicates. Bayesian inference (BI) analysis was conducted using MrBayes v.3.1.2 [49] by Markov chain Monte Carlo sampling (MCMC) based on four simultaneous Markov chains, with 1,000,000 generations and sampling every 100th generation. The first 25% of trees of the burn-in phase were discarded, and the remaining 75% of trees were used for calculating the posterior probability (PP). The phylogenetic trees were customized with FigTree v.1.4.0 [50] and rearranged in Adobe Illustrator CC 22.0.0 (Adobe Systems, San Jose, CA, USA).

## 3. Results

Out of 53 rice samples collected from 12 locations, 39 fungal isolates were obtained, all identified as belonging to the Pleosporales order. Five new species (*Bipolaris chiangraiensis*, *Ophiosphaerella oryzae*, *Paraphaeosphaeria oryzae*, *Pyrenochaetopsis oryzicola*, and *Setophoma oryzicola*) were discovered through micro-morphology and phylogeny analyses, along with six new host records and two new geographical records. 

### Taxonomy and Phylogeny

***Pleosporaceae*** Nitschke

*Pleosporaceae* introduced by Nitschke [51] is the largest family in the Pleosporales and is considered the representative of the order [18,21,22,23,52]. The family includes pathogenic or saprobic species on wood and dead herbaceous stems and leaves, as well as some pathogens in humans [53,54]. The sexual morph of this family is recognized by its perithecial ascomata which is usually black and sometimes setose with bitunicate and cylindrical asci and phragmosporous ascospores [21,55]. The asexual morph can be coelomycetous or hyphomycetous with phialidic, annellidic or sympodial blastic conidiogenous cells. The most common genera are *Alternaria*, *Bipolaris*, and phoma-like genera, which exist as saprobes or parasites on various host plants [23,56].

**Figure 1:**
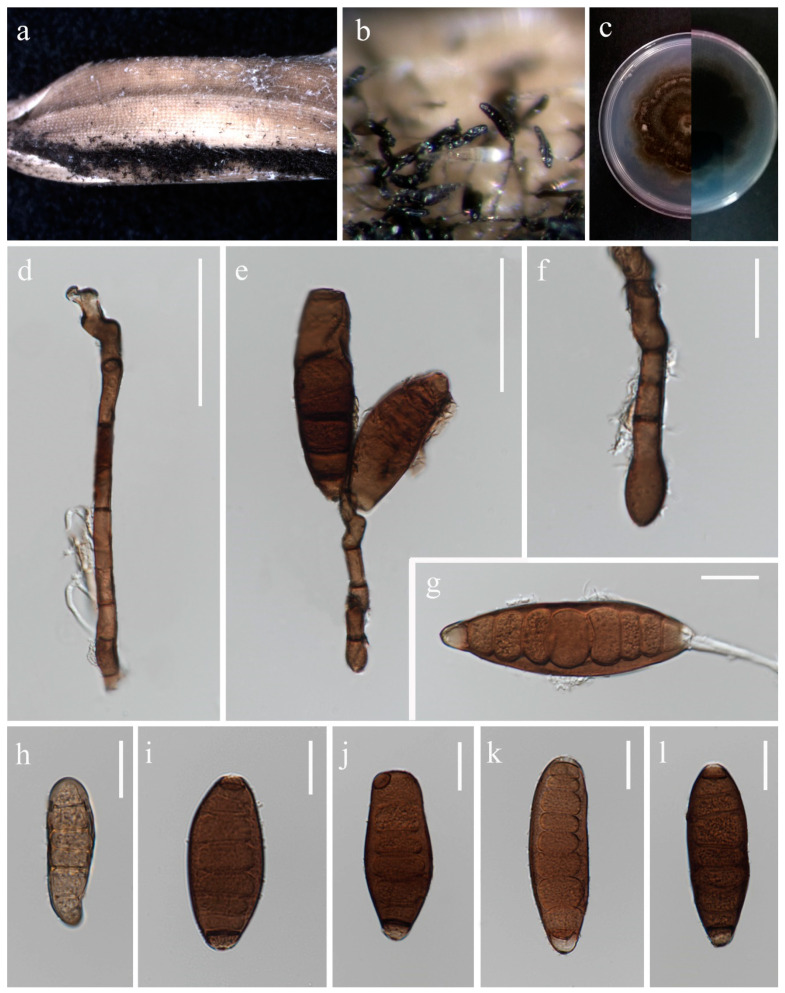
*Bipolaris chiangraiensis* (MFLU 24-0090, holotype). (**a**) Appearance of conidial mass on panicle of Oryza sativa; (**b**) Close-up of conidiophores and conidia on the substrate; (**c**) Top and reverse of colony on PDA; (**d**) Conidiophore; (**e**) Conidiogenous cells and conidia; (**f**) Base of conidiophore; (**g**) Unipolar germinating conidium; (**h**–**l**) Conidia from immature to mature. Scale bars: (**d**–**e**) = 50 μm; (**f**–**l**) = 20 μm.

***Bipolaris chiangraiensis*** S. Absalan, S. Lumyong & K. D. Hyde, sp. nov. (Figure 1).

Index Fungorum number: IF902533; Facesoffungi number: FoF 16746

Etymology: Named after Chiang Rai Province from where it was collected.

*Saprobic* on panicle. **Sexual morph:** Not observed. **Asexual morph:** *Hyphae* 3–5 μm wide, branched, septate, hyaline to pale brown. *Conidiophores* 140–420 × 5.5–9 μm ($\bar{x}$ = 280 × 7 μm, n = 10), septate, mostly simple and straight, infrequently branched, micronematous to semi-macronematous, considerably swollen at apex, brown to dark brown. *Conidiogenous cells* 9–19 × 5–6.5 μm ($\bar{x}$ = 11 × 5.5 μm, n = 6), terminal or intercalary, sympodial, subcylindrical to slightly swollen, pale brown to brown. *Conidia* 54–93 × 12–30 μm ($\bar{x}$ = 70 × 19 μm, n = 20), 5–9-distoseptate, smooth-walled, straight, rarely curved, fusiform, obovoid, sub-cylindrical to broadly swollen, unipolar germination, brown to dark brown, basal cells pale brown.

Culture characteristics: Colonies on PDA reaching 55–58 mm diameter after a week at 27 °C, irregular margin, sparse aerial mycelium, cottony, dark grey to dark olivaceous grey; reverse dark grey.

Material examined: Thailand, Chiang Rai Province, Mueang Chiang Rai District, Tha Sut subdistrict, on panicle of *Oryza sativa*, 17 December 2021, Sahar Absalan, TS102, (MFLU 24-0090, holotype); (ex-type living culture MFLUCC 24-0016).

GenBank numbers: ITS = PQ368326, *tef*1 = PQ412470, *gapdh* = PQ412512

**Figure 2:**
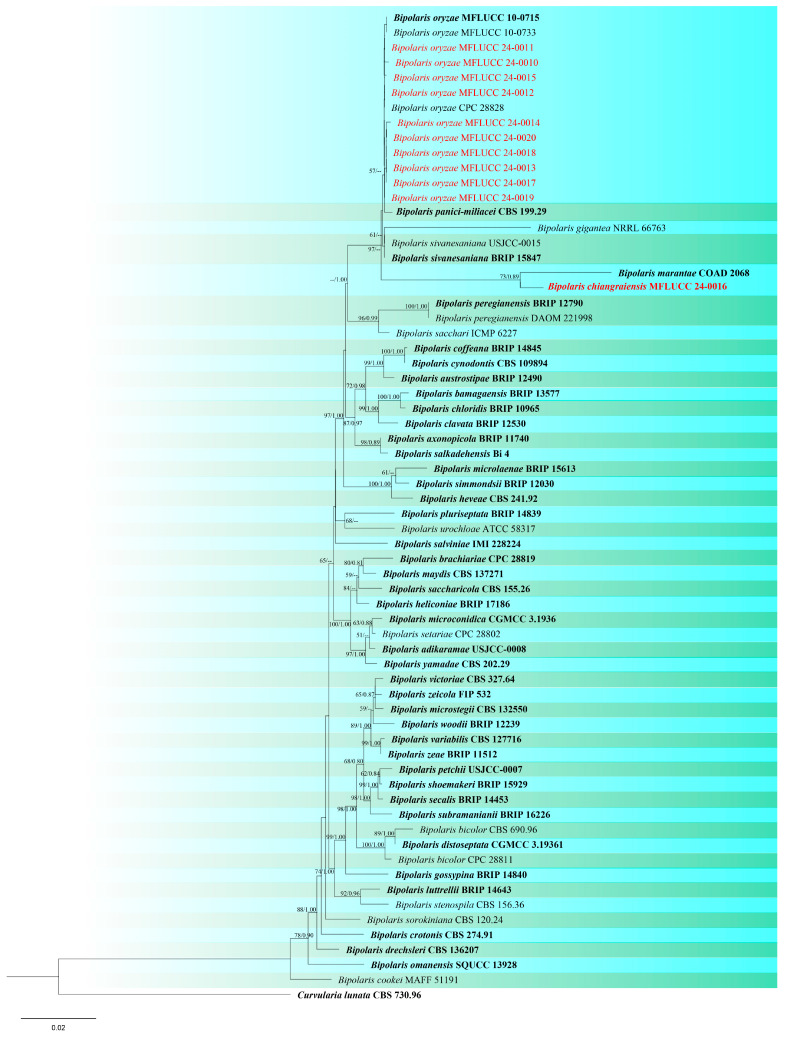
Phylogenetic tree of *Bipolaris* generated from a maximum likelihood analysis based on concatenated ITS, *gapdh*, and *tef*1 sequence data. Sixty-six strains were included in the combined analyses, which comprised 2013 characters after alignment. Nodes on the tree with bootstrap values ≥50% for IQ-Tree and ≥0.7 for Bayesian posterior probabilities (BYPP) are indicated. Isolates from the current study are in red, while ex-type strains are in bold black. The tree is rooted with *Curvularia lunata* (CBS 730.96), and the scale bar represents the expected number of nucleotide substitutions per site.

Notes: According to phylogenetic analyses, isolate MFLUCC 24-0016 is closely related to *Bipolaris marantae* with 73% ML and 0.89 BYPP support (Figure 2). However, pairwise DNA sequence comparison revealed that our strain is distinct with 3% base pair differences across 550 nucleotides in the ITS region and 2% base pair differences across 339 nucleotides in *gapdh* region. The conidia of *B. marantae* are 80–150 × 12.5–22.5 μm, mostly 5–7 distoseptate and bipolar germination. *B. chiangraiensis* has smaller conidia, mostly 5–9 distoseptate, and unipolar germination. *B. marantae*, was isolated from leaf spots on *Maranta leuconeura* (Marantaceae) in Brazil [57].

***Bipolaris oryzae*** (Breda de Haan) Shoemaker, Canadian Journal of Botany 37(5): 883 (1959). (Figure S1).

Index Fungorum number: IF482518; Facesoffungi number: FoF 14290

For description see Manamgoda et al. [29].

Material examined: Thailand, Chiang Rai Province, Mueang Chiang Rai District, Tha Sut subdistrict, on panicle of *Oryza sativa* (Poaceae), 17 December 2021, Sahar Absalan, TS97, (MFLU 24-0089); (living culture MFLUCC 24-0015). See Table 2 for details of other strains.

GenBank numbers: MFLUCC 24-0010: ITS = PQ368316, *tef*1 = PQ412460, *gapdh* = PQ412502; MFLUCC 24-0011: ITS = PQ368317, *tef*1 = PQ412461, *gapdh* = PQ412503; MFLUCC 24-0012: ITS = PQ368318, *tef*1 = PQ412462, *gapdh* = PQ412504; MFLUCC 24-0013: ITS = PQ368319, *tef*1 = PQ412463, *gapdh* = PQ412505; MFLUCC 24-0014: ITS = PQ368320, *tef*1 = PQ412464, *gapdh* = PQ412506; MFLUCC 24-0015: ITS = PQ368321, *tef*1 = PQ412465, *gapdh* = PQ412507; MFLUCC 24-0017: ITS = PQ368322, *tef*1 = PQ412466, *gapdh* = PQ412508; MFLUCC 24-0018: ITS = PQ368323, *tef*1 = PQ412467, *gapdh* = PQ412509; MFLUCC 24-0019: ITS = PQ368324, *tef*1 = PQ412468, *gapdh* = PQ412510; MFLUCC 24-0020: ITS = PQ368325, *tef*1 = PQ412469, *gapdh* = PQ412511

Notes: Ten strains clustered with *B. oryzae* in the combined three-gene analysis. The type strain (MFLUCC 10-0715) was originally isolated from *Oryza sativa* [58], as were all of the strains in this study. There were no nucleotide differences between the type strain and most of our strains, except for isolate MFLUCC 24-0010 with one nucleotide difference in *gapdh*, and one in *tef*1 loci, and isolate MFLUCC 24-0015 and MFLUCC 24-0014 with one and two different nucleotides in *gapdh* locus respectively. The strains were isolated from different varieties of rice collected from four districts, Mueang Chiang Rai, Phan, Mae Sai, and Mae Chan. *Bipolaris oryzae* is best known as a significant phytopathogen [59]. However, we isolated eight strains as saprobes from panicles and stems, and two strains were obtained as endophytes from leaves (Table 2).

***Curvularia chiangmaiensis*** Y. Marín, Senwanna & Crous, in Marin-Felix, et al. Mycosphere 8(9): 1565 (2017). (Figure S2).

Index Fungorum number: IF822082; Facesoffungi number: FoF 12887

For description see Marin-Felix et al. [60].

Material examined: Thailand, Chiang Rai Province, Phan District, Leaf spot on *Oryza sativa*, 16 July 2022, Sahar Absalan, NS169, (MFLU 24-0196); (living culture MFLUCC 24-0024).

GenBank numbers: ITS = PQ358372, *tef*1 = PQ412459, *gapdh* = PQ412487

**Figure 3:**
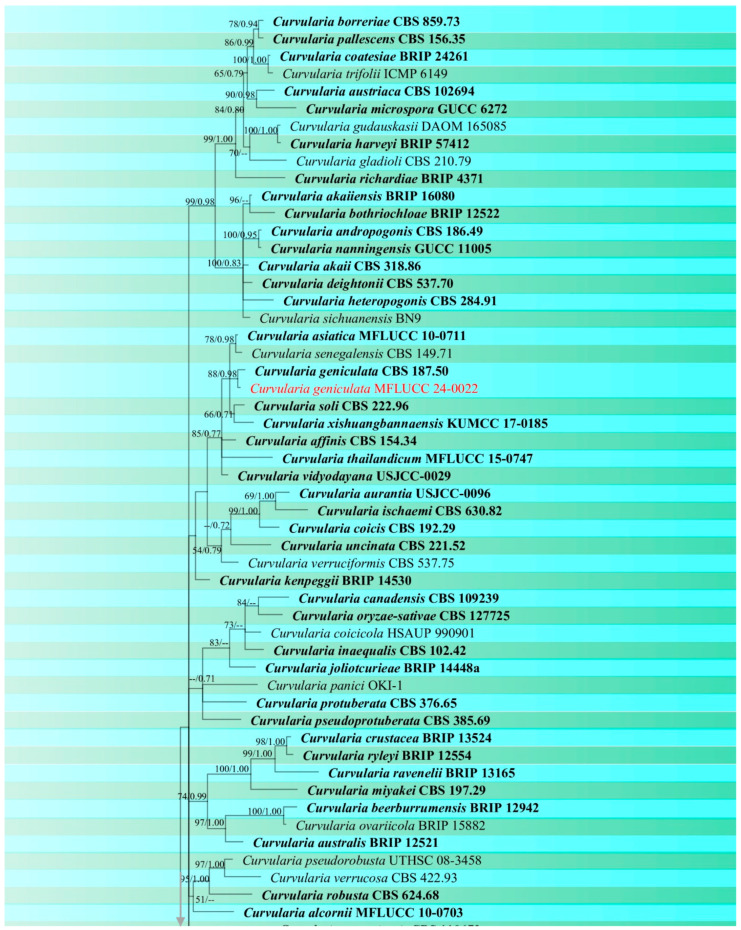

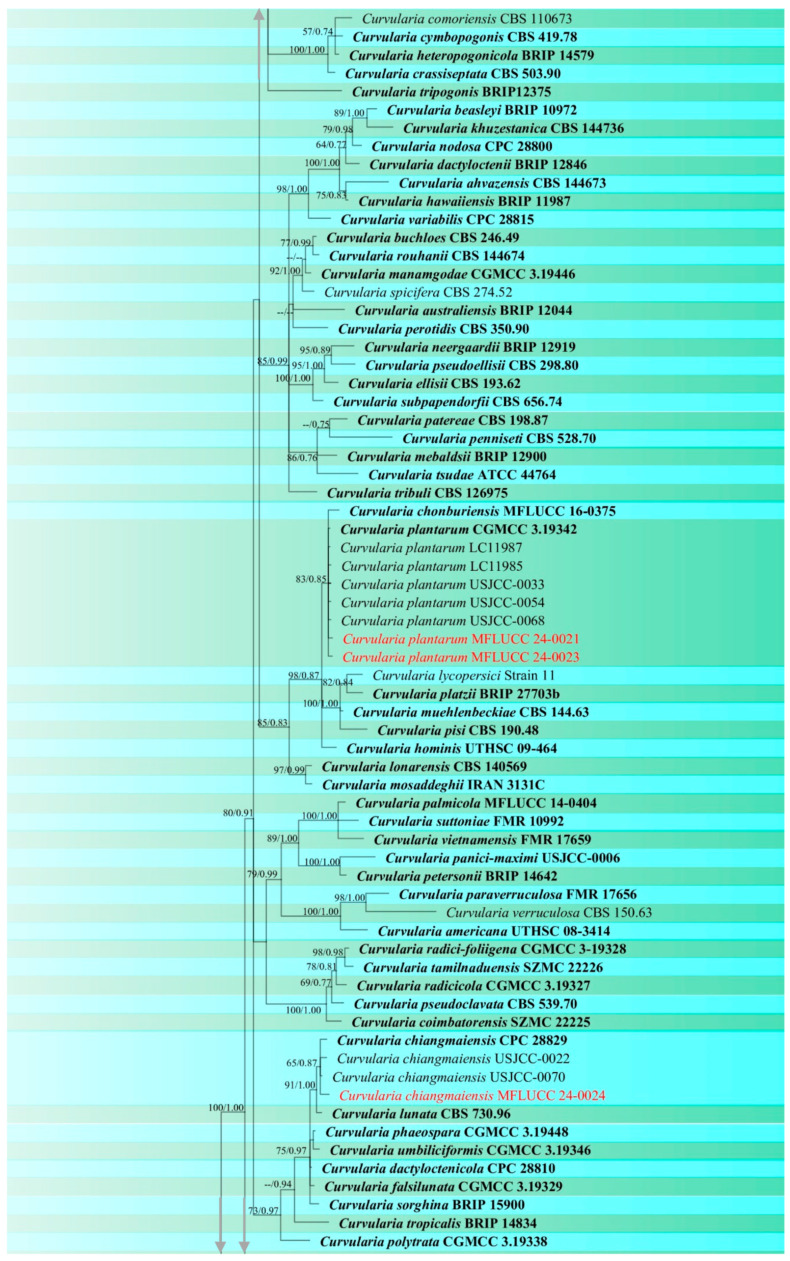

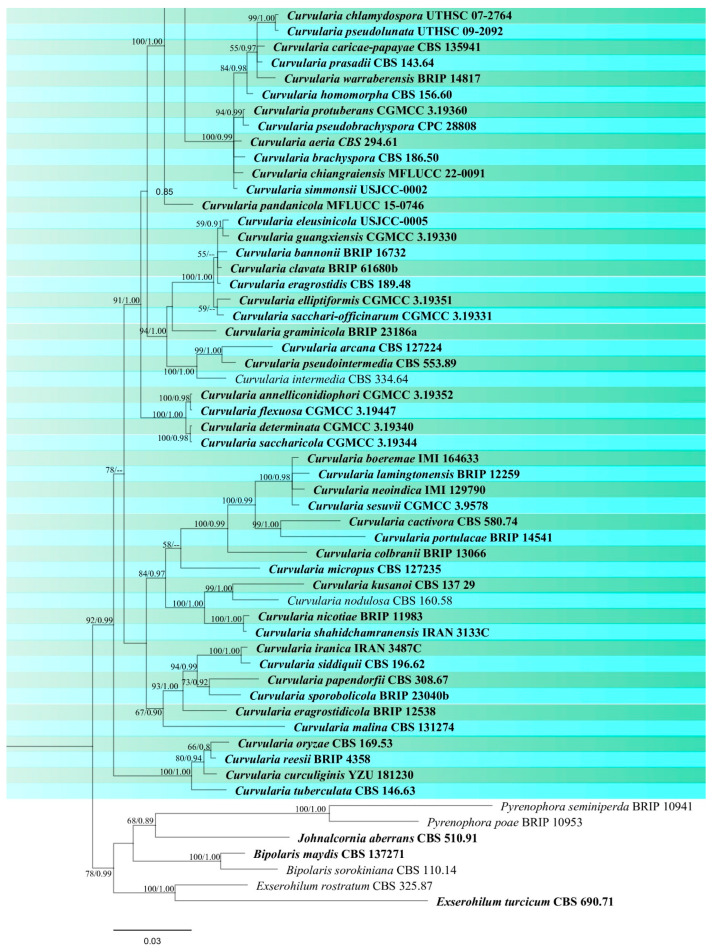
Phylogenetic tree of *Curvularia* generated from a maximum likelihood analysis based on concatenated ITS, *gapdh*, and *tef*1 sequence data. A total of 177 strains were included in the combined analyses, which comprised 1791 characters after alignment. Nodes on the tree supported by bootstrap values ≥50% for IQ-Tree and ≥0.7 for Bayesian posterior probabilities (BYPP) are indicated. Isolates from the current study are in red, while ex-type strains are in bold black. The tree was rooted with other members of *Pleosporaceae* (*Bipolaris maydis*, *B. sorokiniana*, *Exserohilum turcicum*, *E. rostratum*, *Johnalcornia aberrans*, *Pyrenophora poae*, and *P. seminiperda*), and the scale bar represents the expected number of nucleotide substitutions per site.

Notes: Based on phylogeny, our isolate is closely related to the type strain of *Curvularia chiangmaiensis* (CPC 28829) with 65% ML and 0.87 BYPP support (Figure 3). It is challenging to identify most *Curvularia* species from one another by employing solely morphological characteristics. For precise species identification, the use of both morphological and molecular methods should be considered [61,62]. The pairwise DNA sequence comparison and morphological differences were not noticeable; therefore, we identified this isolate as *C. chiangmaiensis* based on phylogenetic analysis and morphological comparison. Our strain (MFLUCC 24-0024) differs slightly from CPC 28829 in the shape and size of conidia [60].

***Curvularia geniculata*** (Tracy & Earle) Boedijn, Bull. Jard. Bot. Buitenzorg 13 (1): 129 (1923). (Figure S3).

Index Fungorum number: IF265873; Facesoffungi number: FoF 16747

For description see Kusai et al. [63].

Material examined: Thailand, Chiang Rai Province, Wiang Chai District, Winag Chai subdistrict, on panicle of *Oryza sativa*, 9 November 2021, Sahar Absalan, NS34-1, (MFLU 24-0092); (living culture MFLUCC 24-0022).

GenBank numbers: ITS = PQ358371, *tef*1 = PQ412458, *gapdh* = PQ412486

Notes: Based on the morphological and molecular data isolate MFLUCC 24-0022 was identified as a representative of *C. geniculata* with 88% ML and 0.98 BYPP support (Figure 3). This species is a cosmopolitan fungus that is most prevalent in tropical countries and can occur on a variety of host plant species [45]. *Curvularia geniculata* is one of the most common *Curvularia* species found on rice and has been reported in various countries such as Bangladesh, Brunei Darussalam, China, India, Japan, Malaysia, Papua New Guinea, Tanzania, and Uganda. [26,64,65,66]. It was reported as a saprobe on wild bananas in Thailand [67], but there are no records from rice.

***Curvularia plantarum*** M. Raza, K. D. Hyde & L. Cai, in Raza, e al., Fungal Diversity 99: 61 (2019). (Figure S4).

Index Fungorum number: IF556664; Facesoffungi number: FoF 06154

For description see Raza et al. [68].

Material examined: Thailand, Chiang Rai Province, Phan District, on the stem of *Oryza sativa*, 28 June 2022, Sahar Absalan, PA155, (MFLU 24-0091); (living culture MFLUCC 24-0023).

GenBank numbers: MFLUCC 24-0021: ITS = PQ358369, *tef*1 = PQ412456, *gapdh* = PQ375101; MFLUCC 24-0023: ITS = PQ358370, *tef*1 = PQ412457, *gapdh* = PQ375102.

Notes: According to the phylogram (Figure 3), isolates MFLUCC 24-0021 and MFLUCC 24-0023 were identified as *Curvularia plantarum* which was originally reported from *Saccharum officinarum* in China [68]. Ferdinandez et al. [44] isolated this species from *Oryza sativa* for the first time from Sri Lanka, which is micro-morphologically more similar to our strains. Our two isolates (MFLUCC 24-0021 and MFLUCC 24-0023) were obtained from panicle spots during the mature stage and stem after harvest, respectively. However, both specimens were collected from the same district (Table 2). *C. plantarum* is reported for the first time from Thailand.

***Phaeosphaeriaceae*** M.E. Barr

*Phaeosphaeriaceae* is one of the largest families in Dothideomycetes [21] comprising 84 genera [22], typified by *Phaeosphaeria*. Barr [69] established *Phaeosphaeriaceae* and delineated the defining characteristics of this family as either saprobic, pathogenic, or hyperparasitic, typically occurring on herbaceous stems, monocotyledonous leaves, culms, or flowers, and occasionally on woody substrates. Members of *Phaeosphaeriaceae* frequently occur on plants in Poaceae [70,71]. The sexual morphs have immersed, erumpent or superficial, globose or conical ascomata with short papilla, small to medium, bitunicate asci, and hyaline, yellow or brown, narrowly or broadly obovoid, aseptate or septate ascospores [18,72]. Asexual morph is characterized by its pycnidial conidiomata which are scattered, solitary or gregarious, immersed to superficial, globose to subglobose, glabrous or setose, light brown to dark brown or black, papillate with enteroblastic or holoblastic, annellidic or phialidic conidiogenous cells. Conidia can vary in shape, hyaline to brown, aseptate or septate, some with appendage or mucilaginous sheath [71,73].

**Figure 4:**
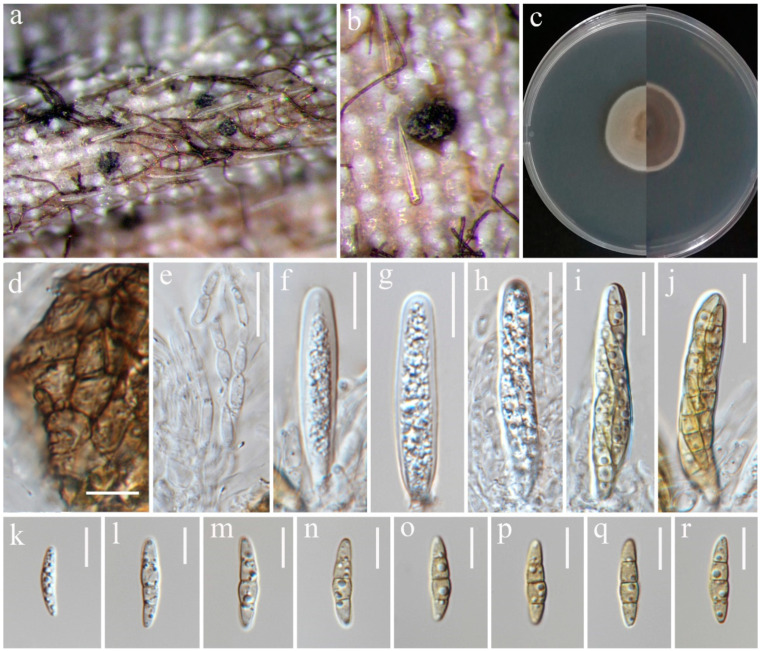
Sexual morph of *Phaeosphaeria musae* (MFLU 24-0316, new host record). (**a**,**b**) Appearance of ascomata on the panicle of *Oryza sativa*; (**c**) Top and reverse of colony on PDA; (**d**) Peridium; (**e**) Pseudoparaphyses; (**f**–**j**) Asci; (**k**–**r**) Ascospores. Scale bars: (**d**–**r**) = 10 μm.

**Figure 5:**
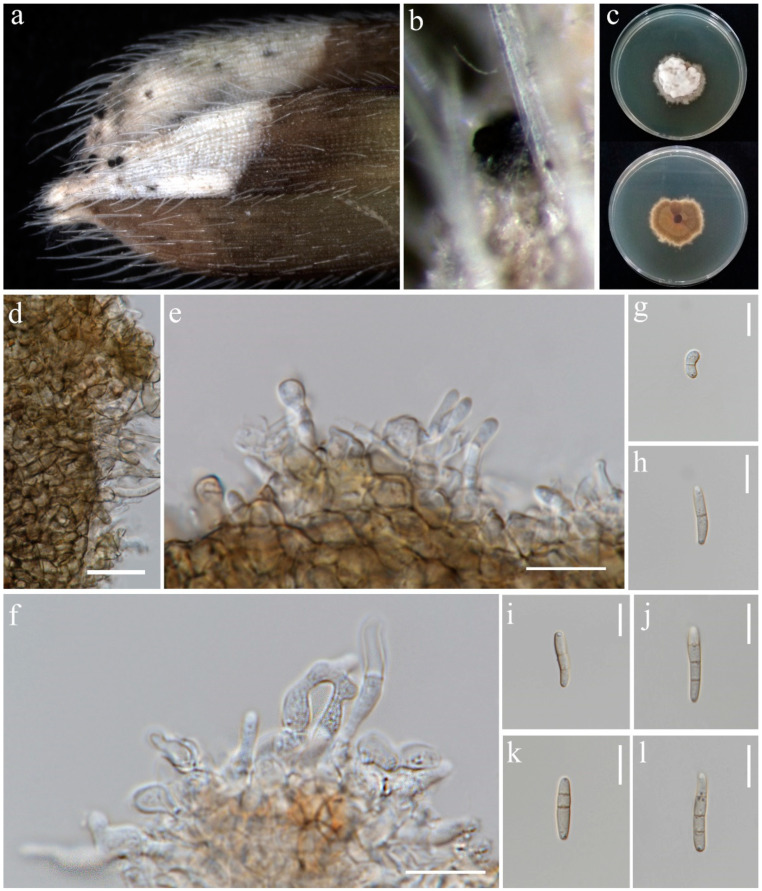
Asexual morph of *Phaeosphaeria musae* (MFLU 24-0100). (**a**,**b**) Appearance of conidiomata on the panicle of *Oryza sativa*; (**c**) Top and reverse of colony on PDA; (**d**) Peridial wall; (**e**,**f**) Conidiogenous cells; (**g**–**l**) Conidia. Scale bars: (**d**) = 20 µm; (**e**–**l**) = 10 µm.

***Phaeosphaeria musae*** Sawada, Special Publication College of Agriculture National Taiwan University 8: 66 (1959). (Figure 4 and Figure 5).

Index Fungorum number: IF336200; Facesoffungi number: FoF00262

*Saprobic* on dead panicle of *Oryza sativa*. **Sexual morph:** *Ascomata* visible as black dots on the surface of the host, scattered, solitary, semi-immersed to superficially raised, ovoid, dark brown to black. *Peridium* 10–17 μm wide, comprising 2–3 layers of brown to dark brown cell walls of *textura angularis*. *Hamathecium* contains numerous 1.5–3 μm wide, cellular, distinctly septate, rarely branching, hyaline pseudoparaphyses. *Asci* 35–57 × 7.5–10.5 μm ($\bar{x}\;$= 44 × 8 μm, n = 20), 8-spored, bitunicate, fissitunicate, cylindrical to slightly clavate, with pointed base, and rounded apex, having indistinct ocular chamber. *Ascospores* 18–23 × 3–5.5 μm ($\bar{x}\;$= 21 × 5 μm, n = 30), fusiform, 3-septate, enlarged at the second cell, slightly constricted at the septa, slightly curved, sometimes straight, rough-walled, yellowish-grey. **Asexual morph:** *Conidiomata* pycnidial, solitary, scattered, most of the structure visible on the surface to semi-immersed, globose to ovoid with ostiole, dark brown to black. *Peridial wall* 15–28 μm wide, comprising several layers of brown, pseudoparenchymatous cells, arranged in a *textura angularis*. *Conidiophores* reduced to conidiogenous cells. *Conidiogenous cells* 2–4 × 3.5–5.5 μm ($\bar{x}\;$= 2.5 × 4.5 μm, n = 10), phialidic, smooth, ampulliform or urceolate, hyaline. *Conidia* 8–22 × 2–3.5 μm ($\bar{x}\;$= 18 × 3 μm, n = 30), solitary, cylindrical, hyaline to pale brown, 1–3-septate, sometimes slightly curved, not constricted at the septa, smooth-walled with guttules.

Culture characteristics: Colonies on PDA reaching 12–13 mm diameter after a week at 27 °C, dense, circular, velvety, pale grey with brown center and yellowish white margin; reverse grey with white margin.

Material examined: Thailand, Chiang Rai Province, Mueang Chiang Rai District, on panicle of *Oryza sativa*, 10 November 2021, Sahar Absalan, RS19-1, (MFLU 24-0316); (living culture MFLUCC 24-0044). Thailand, Chiang Rai Province, Mae Chan District, on panicle of *O. sativa*, 11 April 2022, Sahar Absalan, M2C136, (MFLU 24-0100); (living culture MFLUCC 24-0046).

GenBank numbers: MFLUCC 24-0044: ITS = PQ358508, LSU = PQ373151, SSU = PQ373161, *tef*1 = PQ412471; MFLUCC 24-0045: ITS = PQ358509, LSU = PQ373152, SSU = PQ373162, *tef*1 = PQ412472; MFLUCC 24-0046: ITS = PQ358510, LSU = PQ373153, SSU = PQ373163, *tef*1 = N/A

**Figure 6:**
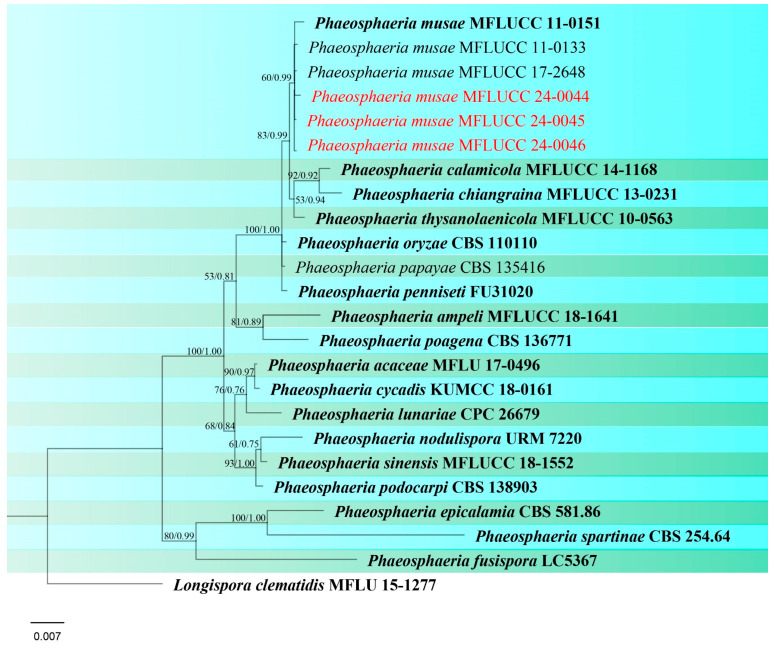
Phylogenetic tree of *Phaeosphaeria* generated from a maximum likelihood analysis based on concatenated ITS, LSU, SSU, and *tef*1 sequence data. A total of 24 strains were included in the combined analyses, which comprised 2986 characters after alignment. Nodes on the tree were supported by bootstrap values, with values ≥50% for RAxML and ≥0.7 for Bayesian posterior probabilities (BYPP). Isolates from the current study are in red, while ex-type strains are in bold black. The tree was rooted with *Longispora clematidis* (MFLU 15-1277), and the scale bar represents the expected number of nucleotide substitutions per site.

Notes: All three strains have a close phylogenetic affinity to the ex-type strain of *Phaeosphaeria musae* (MFLUCC 11-0151) with 60% ML and 0.99 BYPP support (Figure 6). Isolates MFLUCC 24-0044 and MFLUCC 24-0045 were found as sexual morphs and isolated from the post-harvest rice panicle. The asexual morph (strain MFLUCC 24-0046) was isolated from a symptomatic rice panicle (variety Pathum Thani 60) (Table 2). To our knowledge, this is the first report of the asexual morph of *P. musae*, and a new host record from rice. Herein, we establish the sexual–asexual connection of *Phaeosphaeria musae*.

**Figure 7:**
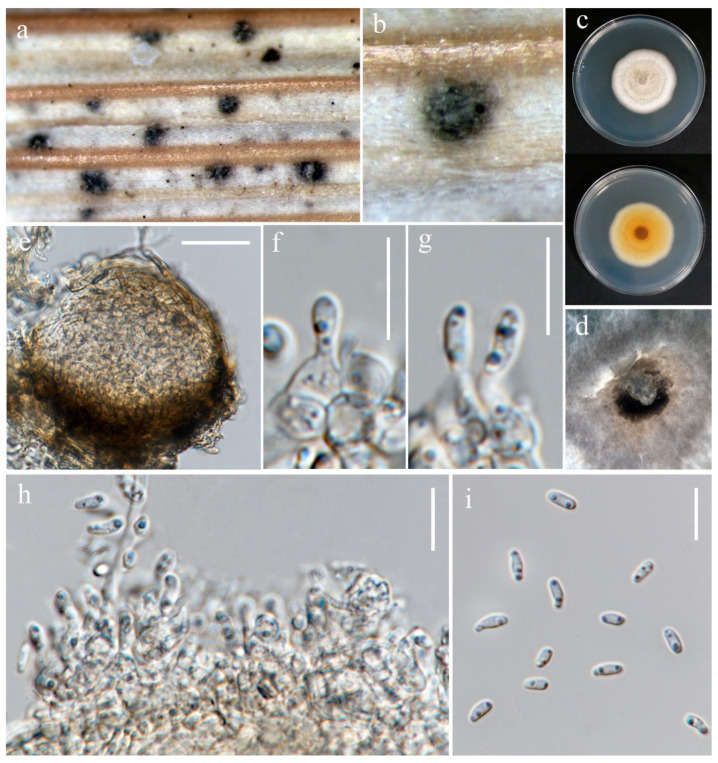
*Setophoma poaceicola* (MFLU 24-0318). (**a**,**b**) Appearance of conidiomata on the stem of *Oryza sativa*; (**c**) Top and reverse of the colony on PDA; (**d**) Conidiomata superficial on PDA after four weeks; (**e**) Section through pycnidium. (**f**–**h**) Conidiogenous cells. (**i**) Conidia. Scale bars: (**e**) = 30 μm; (**f**–**i**) = 20 μm.

***Setophoma poaceicola*** Goonas., Thambug. & K. D. Hyde, Mycosphere 8(4): 763 (2017). (Figure 7).

Index Fungorum number: IF552993; Facesoffungi number: FoF 03212

*Saprobic* on dead stem of *Oryza sativa*. **Sexual morph:** Not observed. **Asexual morph:** *Conidiomata* 90–150 μm diameter, pycnidial, semi-immersed, globose to subglobose, with an opening at apex, with dark brown pycnidial wall. *Conidiophores* reduced to conidiogenous cells lining the inner cavity. *Conidiogenous cells* 3–5 × 2.5–4.5 μm ($\bar{x}\;$= 4 × 4.5 μm, n = 10), hyaline, aseptate, smooth, ampulliform or sometimes irregular. *Conidia* 4–6.5 × 2–2.5 μm ($\bar{x}\;$= 5.5 × 2 μm, n = 30), smooth, cylindrical to oblong, aseptate, rounded at ends, straight or slightly curved, hyaline.

Culture characteristics: Colonies on PDA reaching 30–32 mm diameter after a week at 27 °C, flat with entire edge, circular, fluffy, pale grey in the center, white at the edge; reverse pale brown in the center.

Material examined: Thailand, Chiang Rai Province, Phan District, on stem of *Oryza sativa*, 28 June 2022, Sahar Absalan, PA156, (MFLU 24-0318); (living culture MFLUCC 24-0034).

GenBank numbers: MFLUCC 24-0033: ITS = PQ376618, LSU = PQ376621, *rpb*2 = N/A, *tef*1 = PQ412474; MFLUCC 24-0034: ITS = PQ376619, LSU = PQ376622, *rpb*2 = PQ412500, *tef*1 = PQ412475.

**Figure 8:**
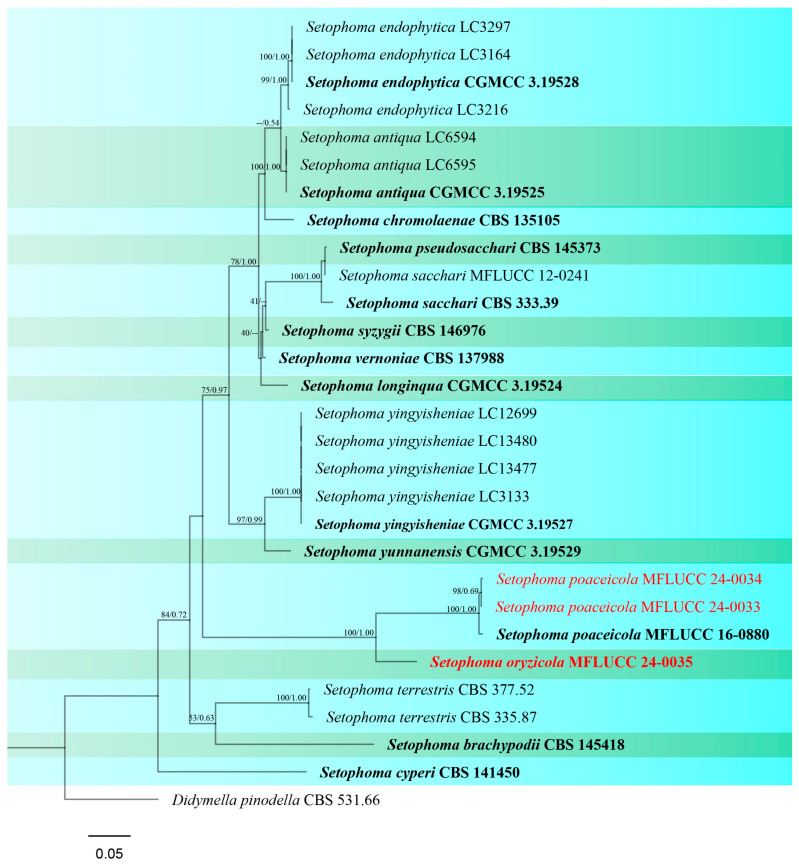
Phylogenetic tree of *Setophoma* generated from a maximum likelihood analysis based on concatenated ITS, LSU, *rpb*2, and *tef*1 sequence data. A total of 29 strains were included in the combined analyses, which comprised 2509 characters after alignment. Nodes on the tree were supported by bootstrap values, with values ≥50% for RAxML and ≥0.7 for Bayesian posterior probabilities (BYPP). Isolates from the current study are in red, while ex-type strains are in bold black. The tree was rooted with *Didymella pinodella* (CBS 531.66), and the scale bar represents the expected number of nucleotide substitutions per site.

Notes: The ex-type strain of *S. poaceicola* (MFLUCC 16–0880) was originally isolated from a dead culm of grass as a saprobic fungus in Thailand [74]. Two strains isolated from Chiang Rai (Wiang Chai and Phan) were identified as *S. poaceicola* with 100% ML and 1.00 BYPP support (Figure 8). Recently, this species was isolated from rice in Indonesia, causing sheath rot [75]. However, our strains were isolated as saprobes from the stem (MFLUCC 24-0034) and panicle (MFLUCC 24-0033) (Table 2). Even though this species was found as a saprobe from Thailand, it may be an emerging pathogen. All studies reported *S. poaceicola* by its sexual morph, and this is the first report of this species in the asexual morph.

**Figure 9:**
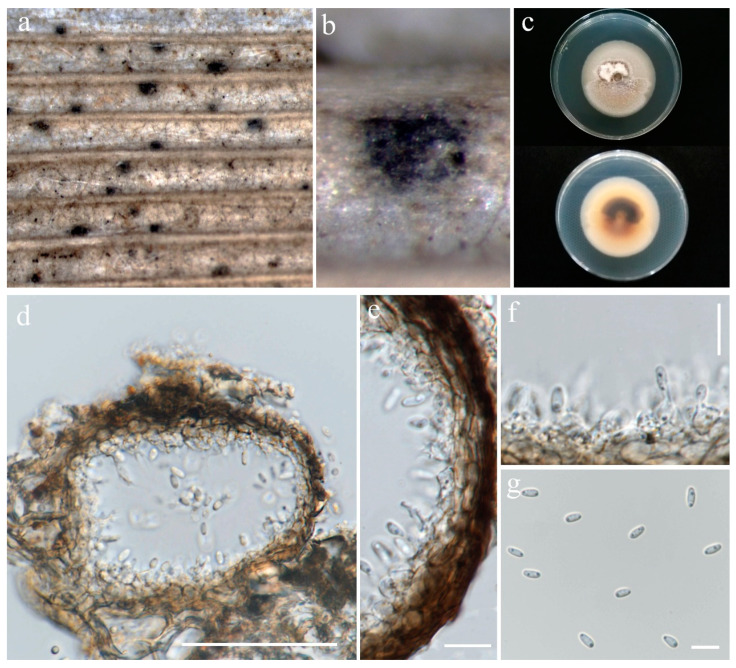
*Setophoma oryzicola* (MFLU 24-0097, holotype). (**a**,**b**) Appearance of conidiomata on the stem of *Oryza sativa*; (**c**) Top and reverse of colony on PDA; (**d**) Section through pycnidium; (**e**) Pycnidial wall; (**f**) Conidiogenous cells; (**g**) Conidia. Scale bars: (**d**) = 50 µm; (**e**,**f**) = 10 µm; (**g**) = 5 µm.

***Setophoma oryzicola*** S. Absalan, S. Lumyong & K. D. Hyde, sp. nov. (Figure 9).

Index Fungorum number: IF902536; Facesoffungi number: FoF 14289

Etymology: Name refers to the host genus *Oryza* from which it was isolated.

*Saprobic* on stem of *Oryza sativa*. **Sexual morph:** Not observed. **Asexual morph:** *Conidiomata* 71–127 μm diameter, 45–76 μm height, pycnidial, scattered, immersed to semi-immersed, globose to subglobose, dark brown to black. *Pycnidial wall* 5–11 μm wide, brown, composed of 3–5 layers of cell walls arranged in a *textura angularis*. *Conidiophores* reduced to conidiogenous cells lining inner cavity. *Conidiogenous cells* 3–5 × 3–5.5 μm ($\bar{x}\;$= 3.5 × 4.5 μm, n = 10), phialidic, hyaline, aseptate, smooth, urceolate to ovoid or sometimes ampulliform. *Conidia* 3.5–6 × 2–3 μm ($\bar{x}\;$= 5 × 2.5 μm, n = 30), smooth, aseptate, ellipsoid to oblong, rounded at ends, with two guttulates, hyaline.

Culture characteristics: Colonies on PDA reaching 16–17 mm diameter after a week at 27 °C, medium dense, irregular, with rhizoid edge, slightly raised, fluffy, white; reverse yellowish-brown.

Material examined: Thailand, Chiang Rai Province, Phan District, on stem of *Oryza sativa*, 28 June 2022, Sahar Absalan, PA157, (MFLU 24-0097, holotype); (ex-type living culture MFLUCC 24-0035).

GenBank numbers: ITS = PQ376620, LSU = PQ376623, *rpb*2 = PQ412501, *tef*1 = PQ412476

Notes: *Setophoma oryzicola* was isolated from a stem of rice as a saprobic fungus (Table 2). In our phylogenetic analyses (Figure 8), *S. oryzicola* is located on a separate branch, forming a well-supported lineage (100% ML and 1.00 BYPP) basal to *S. poaceicola* clade. In a pairwise comparison, *S. oryzicola* and *S. poaceicola* (MFLUCC 16–0880) differ in ITS (15.2%), LSU (1.7%), and RPB2 (24.4%). It is challenging to differentiate between these two species based on morphology. However, conidiogenous cells in *S. poaceicola* are mainly ampulliform, while they are usually urceolate in *S. oryzicola*. Hence, we introduce *S. oryzicola* as a new species based on morphological examination and phylogenetic analysis.

***Ophiosphaerella agrostidis*** Dern., M.P.S. Câmara, N.R. O’Neill, Berkum & M.E. Palm, Mycologia 92(2): 320 (2000). (Figure S5).

Index Fungorum number: IF464614; Facesoffungi number: FoF00258

For description see Câmara et al. [76].

Material examined: Thailand, Chiang Rai Province, Mueang Chiang Rai District, Huai Sak subdistrict, on stem of *Oryza sativa*, 17 December 2021, Sahar Absalan, HS58-1, (MFLU 24-0094); (living culture MFLUCC 24-0029).

GenBank numbers: ITS = PQ374211, LSU = PQ374216, SSU = PQ394981, *tef*1 = PQ412454.

**Figure 10:**
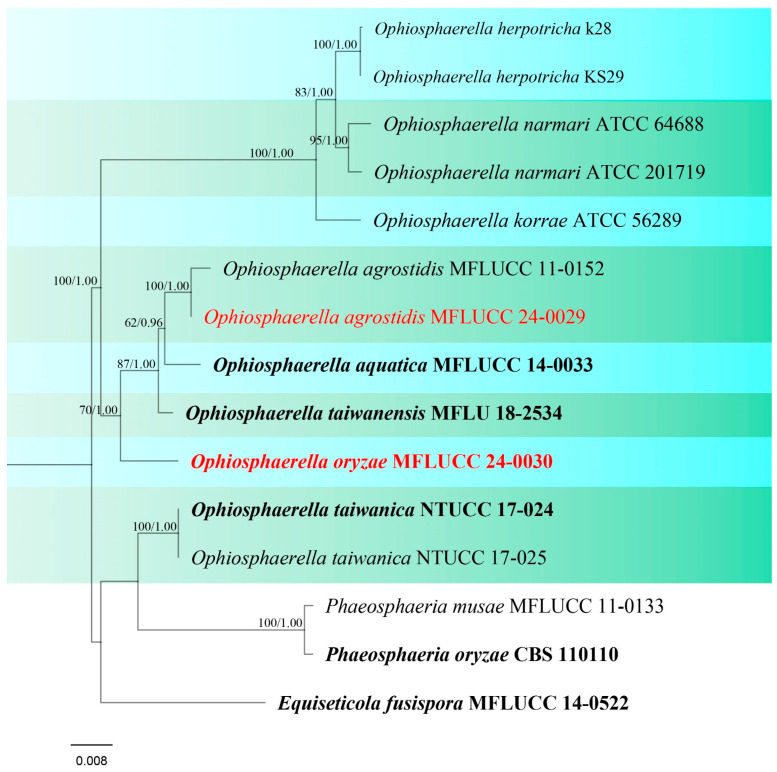
Phylogenetic tree of *Ophiosphaerella* generated from a maximum likelihood analysis based on a concatenated ITS, LSU, SSU, and *tef*1 sequence data. A total of 15 strains were included in the combined analyses, which comprised 3262 characters after alignment. Nodes on the tree were supported by bootstrap values, with values ≥50% for IQ-Tree and ≥0.7 for Bayesian posterior probabilities (BYPP). Isolates from the current study are in red, while ex-type strains are in bold black. The tree was rooted with *Phaeosphaeria musae* (MFLUCC 11-0133), *P. oryzae* (CBS 110110), and *Equiseticola fusispora* (MFLUCC 14-0522), and the scale bar represents the expected number of nucleotide substitutions per site.

Notes: Phylogenetic analyses of concatenated ITS, LSU, SSU, and *tef*1 sequence data showed that our strain, MFLUCC 24-0029, is grouped with *Ophiosphaerella agrostidis* (Figure 10) which was first reported from *Agrostis palustris* [76]. Most of the species within *Ophiosphaerella* are pathogens on various hosts; however, studies in Thailand revealed that *O. agrostidis* can thrive as a saprobe [71,74]. In this study, MFLUCC 24-0029 was isolated from a dead rice plant stem (Table 2), representing a new host record from *Oryza sativa*.

**Figure 11:**
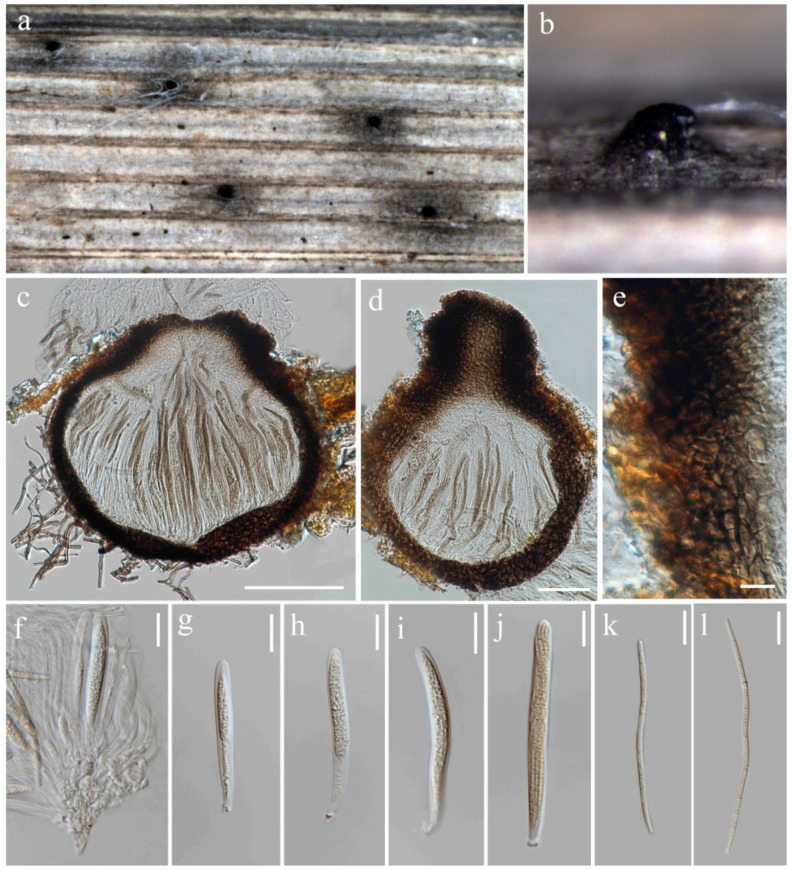
*Ophiosphaerella oryzae* (MFLU 24-0095, holotype). (**a**,**b**) Appearance of ascomata on the stem of *Oryza sativa*; (**c**) Section through ascoma; (**d**) Ascoma with ostiole; (**e**) Peridium; (**f**) Ascogenous cells; (**g**–**j**) Asci; (**k**,**l**) Ascospores. Scale bars: (**c**) = 100 µm; (**d**) = 50 µm; (**e**) = 10 µm; (**f**–**l**) = 20 µm.

***Ophiosphaerella oryzae*** S. Absalan, S. Lumyong & K. D. Hyde, sp. nov. (Figure 11).

Index Fungorum number: IF902534; Facesoffungi number: FoF 16748

Etymology –Refers to the host genus *Oryza* from which it was isolated.

*Saprobic* on dead stem of *Oryza sativa*. **Sexual morph:** *Ascomata* 169–255 μm diameter, solitary, globose to subglobose, semi-immersed with erumpent neck, ostiolate, dark brown to black. *Ostiole* 30–101 × 90–115 μm ($\bar{x}\;$= 83 × 103 μm, n = 5) diameter, papillate. *Peridium* 20–35 μm wide, comprising 4–6 layers of dark brown pseudoparenchymatous cells arranged in a *textura angulari* forming a fairly thick wall. *Asci* 41.5–123 × 6–11.5 μm ($\bar{x}\;$= 83 × 7.5 μm, n = 20), 8-spored, arising from simple aseptate ascogenous cells, bitunicate, cylindrical and sometimes slightly curved, pedunculated, narrower toward the base. *Ascospores* 90–145 × 3–4 μm ($\bar{x}\;$= 118 × 3.5 μm, n = 30), filiform, smooth-walled, with 13 transverse septa, lying parallel or partly spirally twisted in ascus, yellow to pale brown. **Asexual morph:** Not observed.

Culture characteristics: Colonies on PDA reaching 17–18 mm diameter after a week at 27 °C, slightly raised, floccose to cottony, brownish grey with irregular fluffy white edge; reverse brown with irregular white edge.

Material examined: Thailand, Chiang Rai Province, Mueang Chiang Rai District, Huai Sak subdistrict, on stem of *Oryza sativa*, 17 December 2021, Sahar Absalan, HS68-1, (MFLU 24-0095, holotype); (ex-type living culture MFLUCC 24-0030).

GenBank numbers: ITS = PQ374212, LSU = PQ374217, SSU = PQ394982, *tef*1 = PQ412455.

Notes: *Ophiosphaerella oryzae* is proposed as a new species based on the multi-gene phylogenetic analyses and morphology. Our strain (MFLUCC 24-0030), clustered in a clade with other species, comprising *O. agrostidis*, *O. aquatica*, and *O. taiwanensis*, in a separate branch with 70% ML, and 1.00 BYPP support (Figure 10). A comparison of the nucleotide differences between *O. oryzae* and the closely related species, *O. taiwanensis*, revealed 0.46% (across 428 nucleotides) and 6.3% (across 620 nucleotides) base pair differences in ITS and *tef*1 gene regions, respectively. Morphological characters of *Ophiosphaerella oryzae* has smaller ascomata, shorter ostiole, and lacks periphyses in the ostiole when compared to *O. taiwanensis* [77].

***Pyrenochaetopsidaceae*** Valenz.-Lopez, Crous, Cano, Guarro & Stchigel

Valenzuela-Lopez et al. [78] introduced *Pyrenochaetopsidaceae* to accommodate *Pyrenochaetopsis* as the type genus. Based on their phylogenetic studies, *Neopyrenochaetopsis* and *Xenopyrenochaetopsis* were also included in this family. The family was initially characterized by its asexual morph until Mapook et al. [20] reported the sexual morph of *Pyrenochaetopsis* for the first time. The family contains species that have been found in different habitats such as plants, water, soil, cysts of plant-parasitic nematodes, and as opportunistic infections in humans [78].

***Pyrenochaetopsis indica*** (T.S. Viswan.) de Gruyter, Aveskamp & Verkley, Mycologia 102(5): 1077 (2010). (Figure S6).

Index Fungorum number: IF514656; Facesoffungi number: FoF 16503

For description see de Gruyter et al. [79].

Material examined: Thailand, Chiang Rai Province, Phan District, on panicle of *Oryza sativa*, 9 November 2021, Sahar Absalan, NS22-1, (MFLU 24-0197); (MFLUCC 24-0036).

GenBank numbers: MFLUCC 24-0036: ITS = PQ376610, LSU = PQ376624, *rpb*2 = N/A, *tub*2 = PQ412483; MFLUCC 24-0037: ITS = PQ376611, LSU = PQ376625, *rpb*2 = PQ412493, *tub*2 = PQ412484.

**Figure 12:**
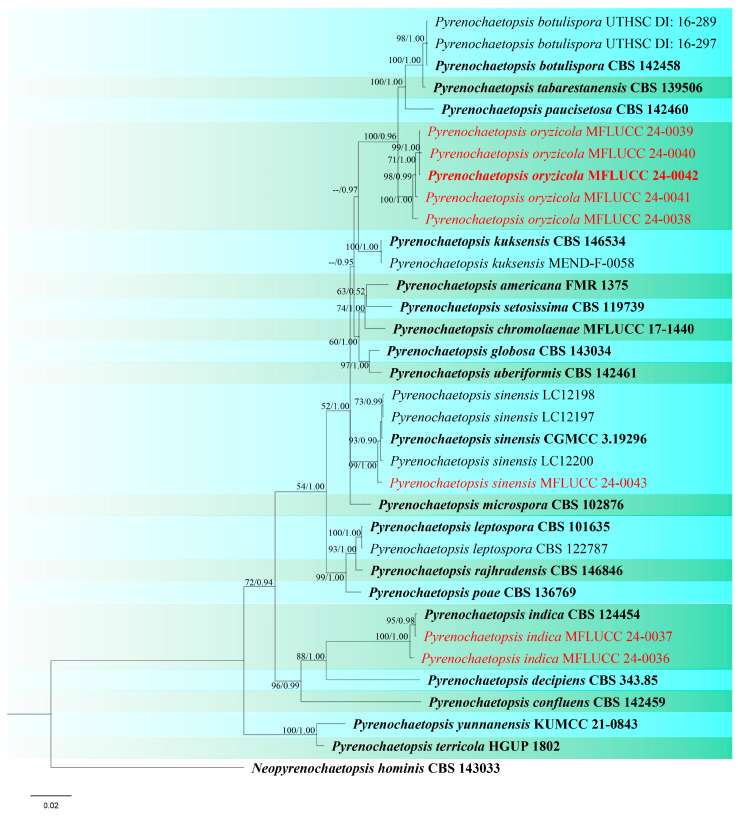
Phylogenetic tree of *Pyrenochaetopsis* generated from a maximum likelihood analysis based on concatenated ITS, LSU, *rpb*2, and *tub*2 sequence data. A total of 35 strains were included in the combined analyses, which comprised 2344 characters after alignment. Nodes on the tree were supported by bootstrap values, with values ≥50% for RAxML and ≥0.7 for Bayesian posterior probabilities (BYPP). Isolates from the current study are in red, while ex-type strains are in bold black. The tree was rooted with *Neopyrenochaetopsis hominis* (CBS 143033), and the scale bar represents the expected number of nucleotide substitutions per site.

Notes: In the combined ITS, LSU, *rpb*2, and *tub*2 phylogeny, our two isolates, MFLUCC 24-0036 and MFLUCC 24-0037, clustered with the ex-type strain of *Pyrenochaetopsis indica* (CBS 124454) with 95% ML, and 0.98 BYPP support (Figure 12). *Pyrenochaetopsis indica* was originally introduced as *Pyrenochaeta indica* by de Gruyter et al. [80], isolated from *Saccharum officinarum* leaves in India. Valenzuela-Lopez et al. [78] presented new genomic sequence data for the ex-type strain of *Pyrenochaeta indica* and introduced a new genus, *Pyrenochaetopsis*, typified by *Pyrenochaetopsis indica*. However, adequate micro-morphological characteristics of this species were not available prior to the present study. We illustrate *P. indica* on rice as a new host record isolated from panicle and stem, collected from two locations in Chiang Rai Province (Table 2).

**Figure 13:**
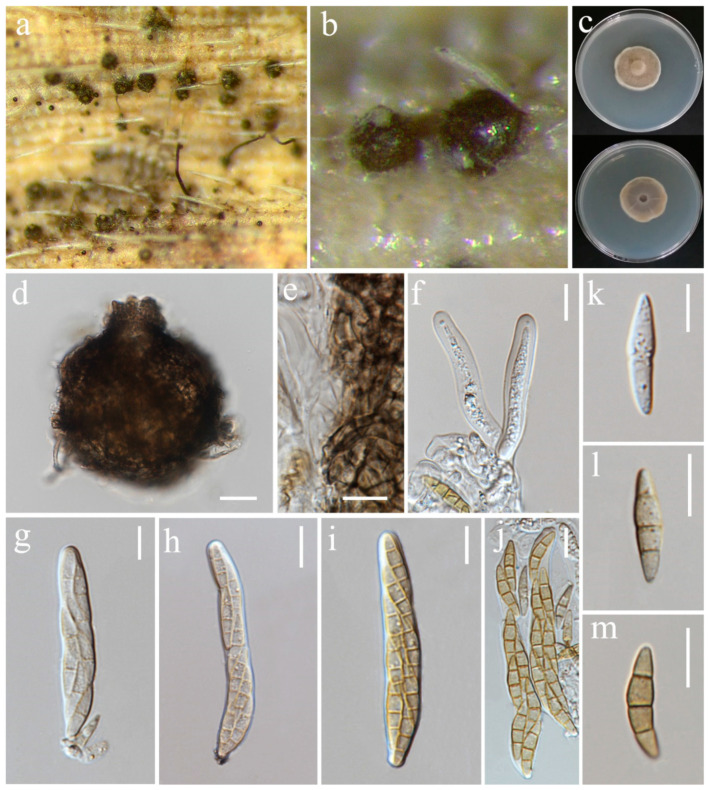
Sexual morph of *Pyrenochaetopsis oryzicola* (MFLU 24-0098, holotype). (**a**,**b**) Appearance of ascomata on the panicle of *Oryza sativa*; (**c**) Top and reverse of colony on PDA; (**d**) Ascoma; (**e**) Peridium; (**f**) Ascogenous cells bearing asci; (**g**–**j**) Asci; (**k**–**m**) Ascospores. Scale bars: (**d**) = 20 µm; (**e**–**m**) = 10 µm.

**Figure 14:**
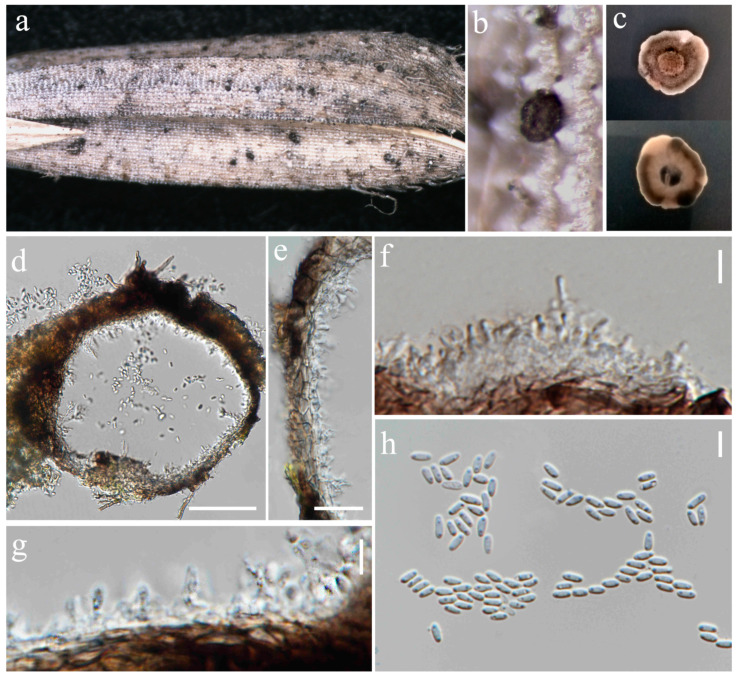
Asexual morph of *Pyrenochaetopsis oryzicola* (MFLU 24-0319, epitype). (**a**,**b**) Appearance of conidiomata on the panicle of *Oryza sativa*; (**c**) Top and reverse of colony on PDA; (**d**) Section through pycnidium; (**e**) Pycnidial wall; (**f**,**g**) Conidiogenous cells; (**h**) Conidia. Scale bars: (**d**) = 50 µm; (**e**) = 10 µm; (**f**–**h**) = 5 µm.

***Pyrenochaetopsis oryzicola*** S. Absalan, S. Lumyong & K. D. Hyde, sp. nov. (Figure 13 and Figure 14).

Index Fungorum number: IF902535; Facesoffungi number: FoF 16749

Etymology: Refers to the host genus *Oryza* from which it was isolated.

*Saprobic* on panicle of *Oryza sativa*. **Sexual morph:** *Ascomata* 76–113 μm diameter, 60–98 μm high, gregarious, scattered to clustered, globose with a short cylindrical opening at apex, mostly visible superficially on the host, sometimes semi-immersed, dark brown to black. *Peridium* 5–11 μm wide, having equal thickness, greyish-brown to brown with several layers of flattened, pseudoparenchymatous cells. *Asci* 42–84 × 7–10 μm ($\bar{x}\;$= 53.5 × 8.5 μm, n = 20), 8-spored, bitunicate, fissitunicate, cylindrical to slightly clavate, with short pedicel, apically rounded. *Ascospores* 16–19 × 3.5–5.5 μm ($\bar{x}\;$= 18 × 4 μm, n = 30), fusiform, slightly curved, 1–3-septate, slightly constricted at central septum, not constricted at other septa, smooth-walled, enlarged at second cell from above, hyaline as immature, becoming shiney yellowish-brown when mature, with small guttules. **Asexual morph:** *Conidiomata* 136–141 μm diameter, 98–116 μm high, visible as raised and superficial on the host, solitary, scattered, sometimes clustered, globosoe to slightly ovoid, dark brown. *Pycnidial wall* 6.5–22 µm wide, composed of several layers of pseudoparenchymatous cells, outer layers pigmented with brown, and becoming paler towards the inner layers. *Conidiophores* reduced to conidiogenous cells. *Conidiogenous cells* 0.5–2.5 × 1–2 μm ($\bar{x}\;$= 1.5 × 1 μm, n = 15), phialidic, hyaline, aseptate, emerging from the cavity inside of the pycnidial wall, difficult to distinguish from the pycnidial wall. *Conidia* 3.5–5 × 1.5–2 μm ($\bar{x}\;$= 4.5 × 2 μm, n = 30), aseptate, cylindrical to ellipsoid, with two guttules, hyaline.

Culture characteristics: Colonies on PDA reaching 31–33 mm diameter after a week at 27 °C, circular, medium dense, cottony, olivaceous grey with pale grey margin; reverse grey.

Material examined: Thailand, Chiang Rai Province, Phan District, on dead panicle of *Oryza sativa*, 9 November 2021, Sahar Absalan, NS28-2a, (MFLU 24-0098, holotype); (ex-type living culture MFLUCC 24-0038). Thailand, Chiang Rai Province, Mueang Chiang Rai District, Tha Sut subdistrict, on dead panicle of *O. sativa*, 17 December 2021, Sahar Absalan, TS110, (MFLU 24-0319, epitype, designated here); (ex-epitype living culture MFLUCC 24-0042).

GenBank numbers: MFLUCC 24-0038: ITS = PQ376612, LSU = PQ376626, *rpb*2 = PQ412494, *tub*2 = PQ412478; MFLUCC 24-0039: ITS = PQ376613, LSU = PQ376627, *rpb*2 = PQ412495, *tub*2 = PQ412479; MFLUCC 24-0040: ITS = PQ376614, LSU = PQ376628, *rpb*2 = PQ412496, *tub*2 = PQ412480; MFLUCC 24-0041: ITS = PQ376615, LSU = PQ376629, *rpb*2 = PQ412497, *tub*2 = PQ412481; MFLUCC 24-0042: ITS = PQ376616, LSU = PQ376630, *rpb*2 = PQ412498, *tub*2 = PQ412482

Notes: According to the phylogenetic analyses, our five strains grouped in a single clade, distinct from closely related species (Figure 12). Three strains (MFLUCC 24-0038, MFLUCC 24-0041, MFLUCC 24-0042) were isolated from the panicle samples, while two strains (MFLUCC 24-0039, MFLUCC 24-0040) were obtained from the stem. Among the isolates, MFLUCC 24-0039, MFLUCC 24-0040, MFLUCC 24-0041, and MFLUCC 24-0042, collected from the Mueang Chiang Rai district, all displayed asexual morphs. In contrast, strain MFLUCC 24-0038, collected from the Phan district, developed a sexual morph (Table 2). *Pyrenochaetopsis oryzicola* is described as a new species based on morphological characteristics of sexual and asexual morphs, as well as phylogenetic analysis of multigene sequence data. We also established the connection between the sexual and asexual stages of this species. Strain MFLU 24-0319 is designated an epitype as it is important to also have authentic material of the asexual morph.

**Figure 15:**
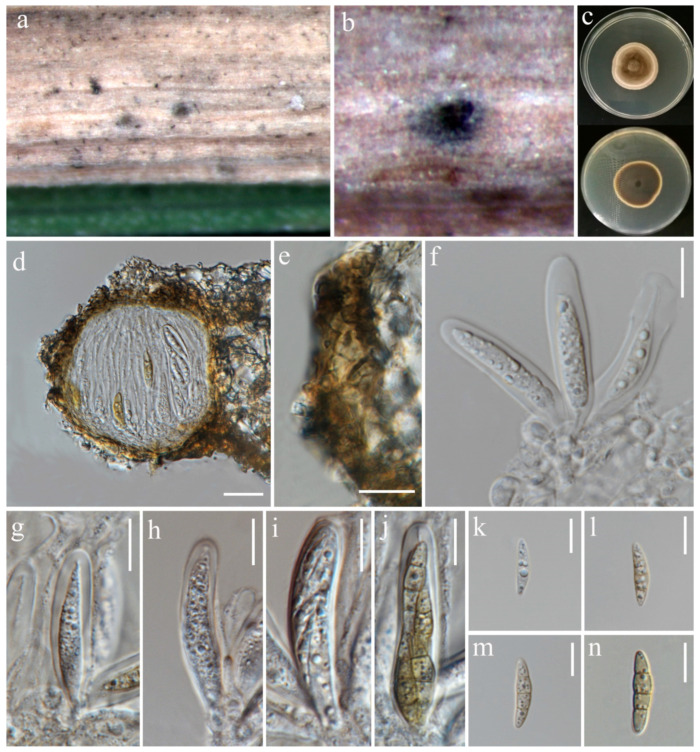
*Pyrenochaetopsis sinensis* (MFLU 24-0099, new host record). (**a**,**b**) Appearance of ascomata on the leaf of *Oryza sativa*; (**c**) Top and reverse of colony on PDA; (**d**) Section through ascoma; (**e**) Peridium; (**f**) Ascogenous cells bearing asci; (**g**–**j**) Asci; (**k**–**n**) Ascospores. Scale bars: (**d**) = 20 µm; (**e**–**n**) = 10 µm.

***Pyrenochaetopsis sinensis*** G.S. Li, J.M. Liang & L. Cai, Fungal Diversity 96: 65 (2019). (Figure 15).

Index Fungorum number: IF556011; Facesoffungi number: FoF 05965

On leaf spots of *Oryza sativa*. **Sexual morph:** *Ascomata* 75–90 μm diameter, 63–70 μm high, solitary, immersed to semi-immersed, globose to sub-globose, dark grey to black, with a dull appearance *Peridium* 13–30 μm wide, thin at the apex, thick at both sides, composed of several layers of pseudoparenchymatous cells, yellowish-brown to hyaline toward the inner layers *Asci* 25–45 × 7–9 μm ($\bar{x}\;$= 36 × 7.5 μm, n = 20), 8-spored, bitunicate, fissitunicate, cylindrical to clavate, rounded at the apex. *Ascospores* 13–23 × 3–4.5 μm ($\bar{x}\;$= 21 × 4 μm, n = 30), fusiform to oblong, 4-septate, second cell from top enlarged, narrower toward the end cells, slightly curved, smooth-walled, hyaline when immature, turning yellowish-grey when mature, with small guttules. **Asexual morph:** Not observed.

Culture characteristics: Colonies on PDA reaching 18–21 mm diameter after a week at 27 °C, circular, cottony, olivaceous grey with yellowish white margin; reverse dark grey with yellowish white margin.

Material examined: Thailand, Chiang Rai Province, Mae Sai District, on leaf spot of *Oryza sativa*, 11 April 2022, Sahar Absalan, M2S120, (MFLU 24-0099); (living culture MFLUCC 24-0043).

GenBank numbers: ITS = PQ376617, LSU = PQ376631, *rpb*2 = PQ412499, *tub*2 = PQ412485

Notes: *Pyrenochaetopsis sinensis* was introduced as a new species by its asexual morph, isolated from soil [81]. In this study, the sexual morph of *P. sinensis* is reported and illustrated for the first time, isolated from leaf spots of *Oryza sativa* (variety: RD-MAEJO2) (Table 2). In the phylogenetic analysis our strain grouped with the ex-type (CGMCC 3.19296) and three strains of *P. sinensis* (Figure 12). The sexual morph of closely related species is unavailable for morphological comparison. Herein, *P. sinensis* is reported as a new host record and its sexual morph is illustrated for the first time.

***Didymellaceae*** Gruyter, Aveskamp & Verkley

de Gruyter et al. [82] established the *Didymellaceae* to include the three genera *Ascochyta*, *Didymella* (type genus), and *Phoma* as well as other related phoma-like species that were classified in this family. *Didymellaceae* contains 44 genera [22], found in a wide range of habitats worldwide. Most members are plant pathogens that affect a variety of hosts and mostly cause lesions on leaves and stems [83,84].

***Epicoccum catenisporum*** Valenz.-Lopez, Stchigel, Crous, Guarro & J.F. Cano, Studies in Mycology 90: 30 (2017). (Figure S7).

Index Fungorum number: IF819762; Facesoffungi number: FoF 16706

For description see Valenzuela-Lopez et al. [78].

Material examined: Thailand, Chiang Rai Province, Wiang Chiang Rung District, on leaf spot of seedling of *Oryza sativa*, 4 July 2022, Sahar Absalan, WCR164, (MFLU 24-0093); (living culture MFLUCC 24-0028).

GenBank numbers: MFLUCC 24-0025: ITS = PQ358494, LSU = PQ358538, *rpb*2 = PQ412489; MFLUCC 24-0027: ITS = PQ358495, LSU = PQ358539, *rpb*2 = PQ412490; MFLUCC 24-0028: ITS = PQ358496, LSU = PQ358540, *rpb*2 = PQ412491

Notes: *Epicoccum catenisporum* (CBS 181.80, ex-type) was formerly identified as *Phoma sorghina*, isolated from leaf spots of rice in Guinea-Bissau [78,85]. Three isolates (MFLUCC 24-0025, MFLUCC 24-0027, MFLUCC 24-0028) collected from three locations in Chiang Rai are phylogenetically related to *E. catenisporum*. Of these, two strains (MFLUCC 24-0025 and MFLUCC 24-0028) were isolated from leaf spots on seedlings, including two different rice varieties (Pathum Thani 60 and RD6). MFLUCC 24-0027 was obtained from a dead stem collected from Phan district as a saprobic fungus (Table 2). This is a new geographical record of *Epicoccum catenisporum* from Thailand.

***Epicoccum latusicollum*** Q. Chen, Crous & L. Cai, Studies in Mycology 87: 144. (Figure S8).

Index Fungorum number: IF818960; Facesoffungi number: FoF 06580

For description see Chen et al. [86].

GenBank numbers: ITS = PQ358497, LSU = PQ358541, *rpb*2 = PQ412492

**Figure 16:**
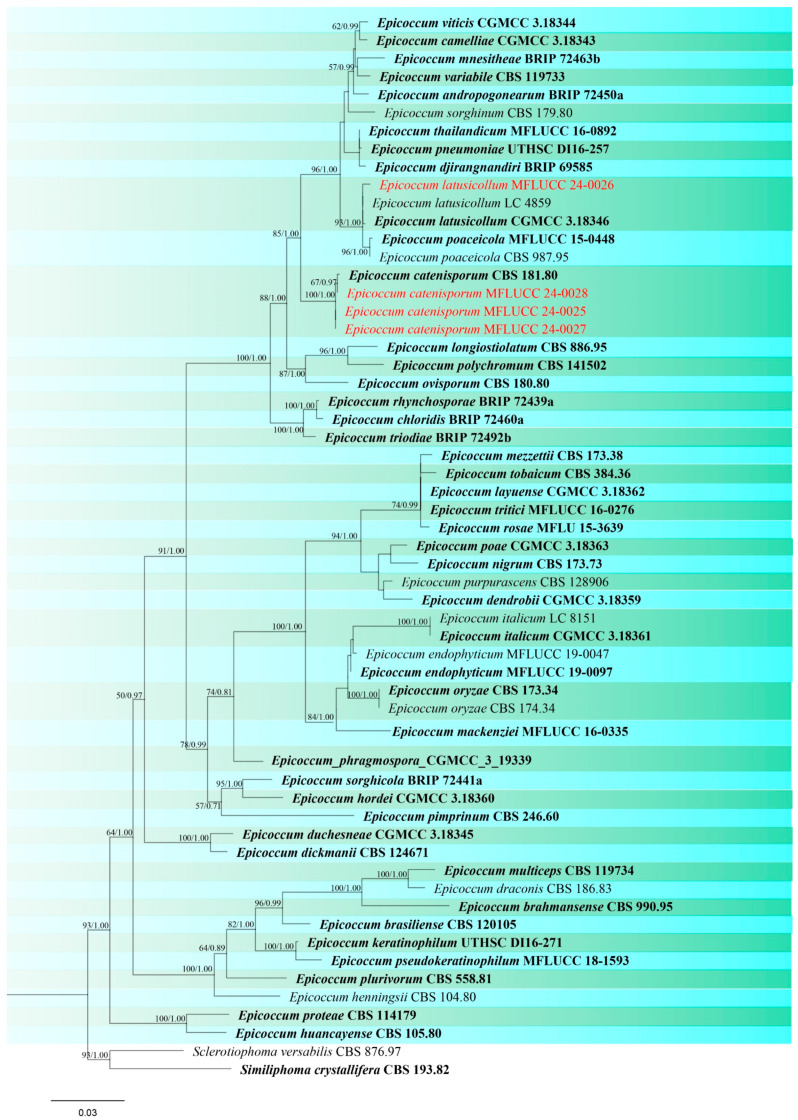
Phylogenetic tree of *Epicoccum* generated from a maximum likelihood analysis based on concatenated ITS, LSU, and *rpb*2 sequence data. A total of 58 strains were included in the combined analyses, which comprised 1993 characters after alignment. Nodes on the tree were supported by bootstrap values, with values ≥50% for RAxML and ≥0.7 for Bayesian posterior probabilities (BYPP). Isolates from the current study are in red, while ex-type strains are in bold black. The tree was rooted with *Sclerotiophoma versabilis* (CBS 876.97) and *Similiphoma crystallifera* (CBS 193.82), and the scale bar represents the expected number of nucleotide substitutions per site.

Notes: Our isolate (MFLUCC 24-0026) clustered with *Epicoccum latusicollum* (CGMCC 3.18346, ex-type) with 93% ML, and 1.00 BYPP support in the combined ITS, LSU, and *rpb*2 phylogenetic analysis (Figure 16). The type of *E. latusicollum* was reported from leaves of *Sorghum bicolor* in China. Other hosts associated with this species include leaves of *Vitex negundo* and *Camellia sinensis* (as an endophyte) in China, and *Podocarpus macrophyllus* in Japan [86]. In our study, this species was isolated from rice leaves collected in Mae Chan district of Chiang Rai, Thailand, and is reported here as a new host record based on phylogenetic analysis and morphological assessment.

***Remotididymella capsici*** (Bond.-Mont.) L.W. Hou, L. Cai & Crous, Studies in Mycology 96: 337 (2020). (Figure S9).

Index Fungorum number: IF834117; Facesoffungi number: FoF 16497

Material examined: Thailand, Chiang Rai Province, Wiang Chai District, endophytic from seedling of *Oryza sativa*, 4 July 2022, Sahar Absalan, WC167a, (living culture MFLUCC 24-0031).

GenBank numbers: ITS = PQ358526, LSU = PQ373118, *rpb*2 = PQ412488

**Figure 17:**
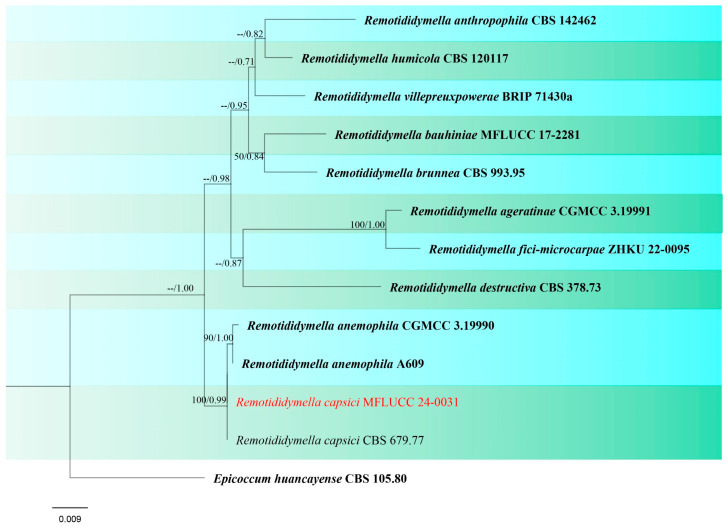
Phylogenetic tree of *Remotididymella* generated from a maximum likelihood analysis based on concatenated ITS, LSU, and *rpb*2 sequence data. A total of 13 strains were included in the combined analyses, which comprised 1570 characters after alignment. Nodes on the tree were supported by bootstrap values, with values ≥50% for RAxML and ≥0.7 for Bayesian posterior probabilities (BYPP). The isolate from the current study is in red, while ex-type strains are in bold black. The tree was rooted with *Epicoccum huancayense* (CBS 105.80), and the scale bar represents the expected number of nucleotide substitutions per site.

Notes: *Remotididymella capsici* was introduced by Hou et al. [87] isolated from leaf spots on *Capsicum annuum* in Fiji, which was originally identified as *Ascochyta capsici*. However, phylogenetic studies revealed that the ex-type strain (CBS 679.77) was clustered in *Remotididymella* resulting in a separate lineage from other species (Figure 17). Morphological characteristics do not exist since their culture remained sterile. In this study, we obtained one strain identified as *R. capsici*, which was isolated as an endophytic fungus from a symptomless seedling a new host record of rice and new geographical record from Thailand. The isolate also did not produce any fruiting body in culture.

***Didymosphaeriaceae*** Munk

*Didymosphaeriaceae* was introduced by Munk [88] and encompasses 33 genera [22]. *Didymosphaeria* Fuckel is considered the type genus of *Didymosphaeriaceae*. The family includes species that are saprotrophic, endophytic, and pathogenic occurring on a wide range of substrates such as wood and herbaceous stems, pods, leaves, and soil [54,89,90]. The sexual morph of *Didymosphaeriaceae* is characterized by globose to subglobose ascomata with central ostiole; cellular or trabeculate pseudoparaphyses; 2–4-spored or 8-spored, bitunicate, pedicellate asci; 1–3-septate or muriform ascospores which are pigmented and in ellipsoid or oblong shapes. The asexual morph can be fusicladium-like and phoma-like [18,21].

***Pseudopithomyces chartarum*** (Berk. & M.A. Curtis) Jun F. Li, Ariyaw. & K. D. Hyde. (Figure S10).

Index Fungorum number: IF551393; Facesoffungi number: FoF 00938

For description see Ariyawansa et al. [91].

Material examined: Thailand, Chiang Rai Province, Wiang Chiang Rung District, on leaf spot of *Oryza sativa*, 4 July 2022, Sahar Absalan, WCR162, (MFLU 24-0101); (living culture MFLUCC 24-0048).

GenBank numbers: MFLUCC 24-0047: ITS = PQ358511, LSU = PQ380128, SSU = PQ380126, *tef*1 = N/A; MFLUCC 24-0048: ITS = PQ358512, LSU = PQ380129, SSU = PQ380127, *tef*1 = PQ412473.

**Figure 18:**
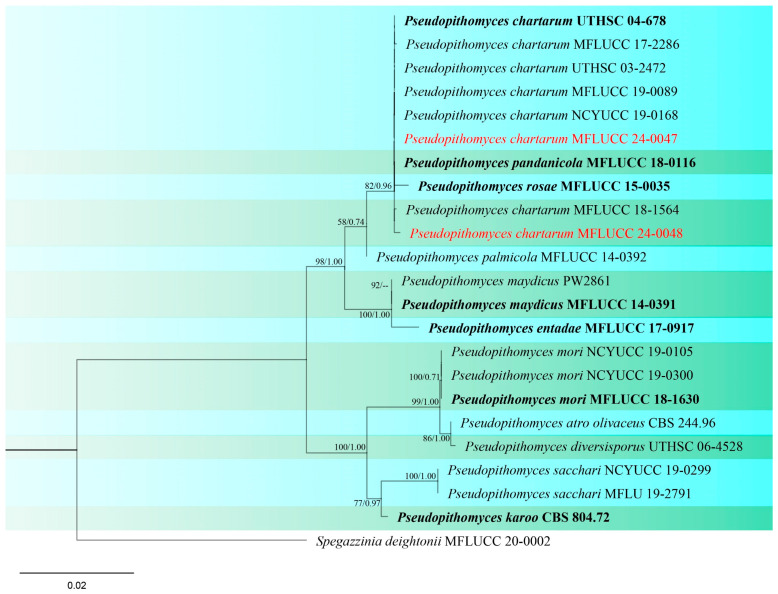
Phylogenetic tree of *Pseudopithomyces* generated from a maximum likelihood analysis based on concatenated ITS, LSU, SSU, and *tef*1 sequence data. A total of 23 strains were included in the combined analyses, which comprised 3009 characters after alignment. Nodes on the tree were supported by bootstrap values, with values ≥50% for IQ-Tree and ≥0.7 for Bayesian posterior probabilities (BYPP). Isolates from the current study are in red, while ex-type strains are in bold black. The tree was rooted with *Spegazzinia deightonii* (MFLUCC 20-0002), and the scale bar represents the expected number of nucleotide substitutions per site.

Notes: The first occurrence of *Pseudopithomyces chartarum* (formerly known as *Pithomyces chartarum*) on rice was reported in India, causing glume blotch of rice [92]. Phylogenetic analyses of concatenated ITS, LSU, SSU, and *tef*1 sequence datasets indicated that our two strains (MFLUCC 24-0047 and MFLUCC 24-0048) have a close affinity with *Pseudopithomyces chartarum* with 82% ML, 0.96 BYPP support (Figure 18). Therefore, based on similarity in the morphological characteristics and phylogenetic analysis, we identified the strains as *P. chartarum*. The two strains were isolated from two different parts of the rice plant, panicle (rice cultivar: RD15) and leaf (rice cultivar: RD6), collected from two districts (Phan and Wiang Chiang Rung) in Chiang Rai Province (Table 2).

**Figure 19:**
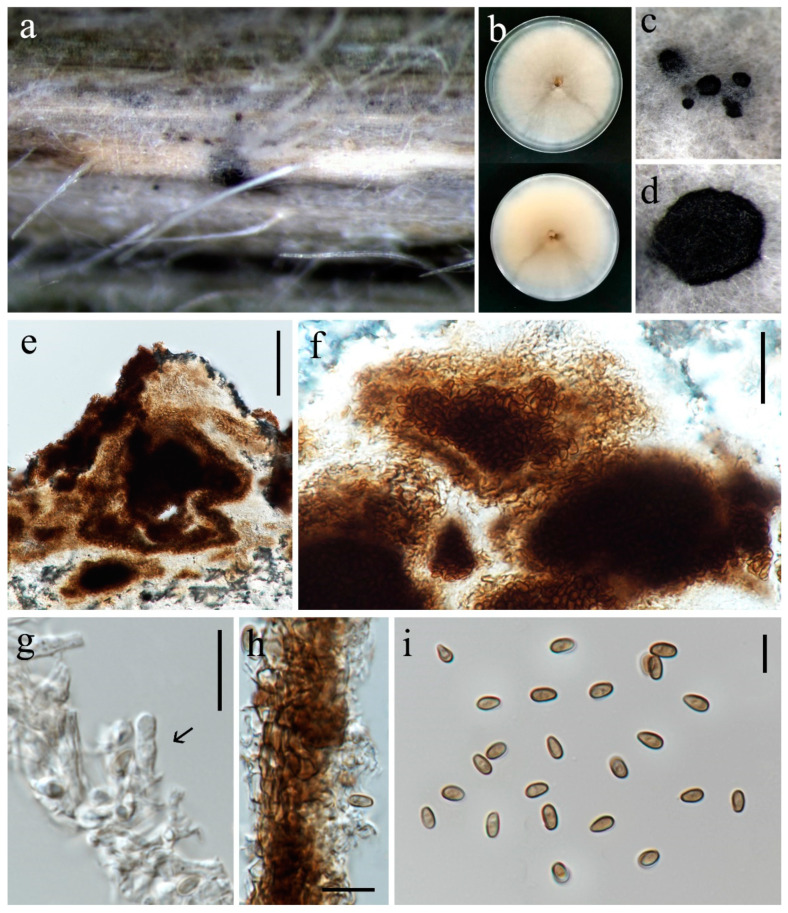
*Paraphaeosphaeria oryzae* (MFLU 24-0096, holotype). (**a**) Appearance of conidiomata on leaf of *Oryza sativa*; (**b**) Top and reverse of colony on PDA; (**c**,**d**) Conidiomata superficial on PDA after three weeks; (**e**,**f**) Cut through the pycnidium; (**g**) Conidiogenous cell (arrow); (**h**) Pycnidial wall; (**i**) Conidia. Scale bars: (**e**) = 50 μm; (**f**–**h**) = 20 μm; (**g**) = 10 μm; (**i**) = 5 μm.

***Paraphaeosphaeria oryzae*** S. Absalan, S. Lumyong & K. D. Hyde, sp. nov. (Figure 19).

Index Fungorum number: IF902539; Facesoffungi number: FoF 16750

Etymology: Name refers to the genus *Oryza* from which it was isolated

On a leaf spot of *Oryza sativa*. **Sexual morph:** Not observed. **Asexual morph:** *Conidiomata* 100–120 μm diameter, pycnidial, solitary, scattered, immersed on the host, and superficial in agar, globose to subglobose. *Pycnidial wall* 14–20 μm wide, comprising several layers of irregular shaped, relatively thick-walled cells of *textura angularis*, pale brown to brown. *Conidiophores* reduced to conidiogenous cells. *Conidiogenous cells* 5–9 × 3–5.5 µm ($\bar{x}\;$= 6 × 4 μm, n = 10), holoblastic, hyaline, doliiform, discrete or lining on mass of cells that are clumped together and protrude into the conidiomatal cavity. *Conidia* 4.5–6 × 2.5–3.5 µm ($\bar{x}\;$= 5 × 3 μm, n = 30) ellipsoid to ovoid, aseptate, smooth, thin-walled, initially hyaline, becoming greyish brown.

Culture characteristics: Colonies on PDA reaching 86–89 mm diameter after a week at 27 °C, circular, slightly fluffy to floccose aerial mycelia, dull white; reverse white.

Material examined: Thailand, Chiang Rai Province, Wiang Chiang Rung District, on leaf spot of *Oryza sativa*, 4 July 2022, Sahar Absalan, WCR161, (MFLU 24-0096, holotype); (ex-type living culture MFLUCC 24-0032).

GenBank numbers: ITS = PQ374213, LSU = PQ374215, *tub*2 = PQ412477

**Figure 20:**
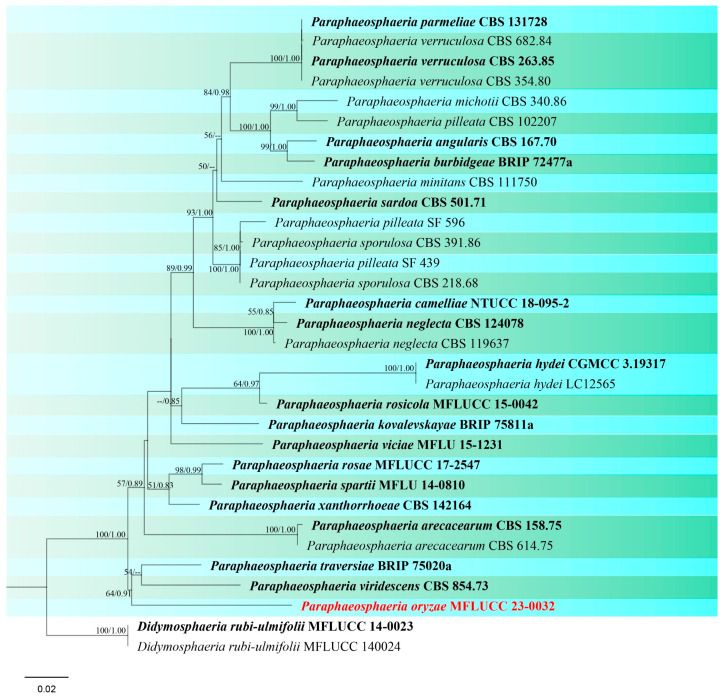
Phylogenetic tree of *Paraphaeosphaeria* generated from a maximum likelihood analysis based on concatenated ITS, LSU, and *tub*2 sequence data. A total of 32 strains were included in the combined analyses, which comprised 1885 characters after alignment. Nodes on the tree were supported by bootstrap values, with values ≥50% for IQ-Tree and ≥0.7 for Bayesian posterior probabilities (BYPP). The isolate from the current study is in red, while ex-type strains are in bold black. The tree was rooted with *Didymosphaeria rubi-ulmifolii* (MFLUCC 14-0023 and MFLUCC 14-0024), and the scale bar represents the expected number of nucleotide substitutions per site.

Notes: Based on combined multi-gene phylogenetic analysis, isolate (MFLUCC 24-0032) clustered as a separate lineage, basal to *Paraphaeosphaeria traversiae* and *P. viridescens* with 64% ML and 0.91 BYPP support (Figure 20). The ex-type of *P. viridescens* (CBS 854.73) was isolated from fresh water in Montenegro [89], while the ex-type of *P. traversiae* (BRIP 75020a) was isolated from a leaf spot of *Cyperus aromaticus* in Australia [93]. Morphologically, *P. oryzae* differs from *P. viridescens* in the color and size of the pycnidial wall (brown, 14–20 μm in *P. oryzae* vs. pale orange, 5–12 µm in *P. viridescens*) and conidia (greyish brown, 5 × 3 μm in *P. oryzae* vs. greenish yellow, 4–4.5 × 1.8–2.2 in *P. viridescens*). Furthermore, *P. viridescens* produces a green pigment in the culture media, while this pigment production is absent in *P. oryzae*. Thus, according to macro- and micro-morphology differences and pairwise sequence comparison (8.7% nucleotide differences across 509 nucleotides in ITS, 1.4% across 844 nucleotides in LSU, 11.7% across 314 nucleotides in *tub*2) we propose this taxon as a new species.

## 4. Discussion

Pleosporales is an order with two suborders, Pleosporineae and Massarineae, containing a quarter of all dothideomycetous species [18,94]. In the current study, all five discovered fungal families belong to the suborder Pleosporineae, which includes many economically important plant pathogen genera with huge species diversity [95]. There are several genera in Pleosporales known as graminicolous fungi such as *Curvularia*, *Bipolaris*, and phoma-like playing important roles in plant diseases. However, many studies have shown that these fungal pathogens can also exist in different nutrient modes as endophytes and saprobes [58,96]. This study also showed that the species belonging to Pleosporales have diverse life modes, thriving as pathogenic, saprobic and endophytic in *Oryza sativa* (Table 2).

*Pleosporaceae*, recognized as the representative family of Pleosporales, is the largest family in the order with 23 accepted genera [21,22]. *Bipolaris* and *Curvularia* are two significant hyphomycetous genera in the family known by their asexual morph [58,97,98,99,100]. Species of *Bipolaris* and *Curvularia* are distributed worldwide and are associated with a wide range of hosts (more than 60 host genera) especially grasses and cereals [26,101,102]. Many species of *Bipolaris* and *Curvularia* have been reported from Thailand. Marin-Felix et al. [60] reported one new species and five new host records of *Bipolaris* during their investigation in Chiang Mai Province. Morphologically, strains of *B. oryzae* isolated in our study have the highest resemblance to strains reported by Marin-Felix et al. [60], in contrast to strains reported from other countries. *Bipolaris oryzae* was the dominant species in our study, with ten strains isolated from different parts of rice. *Curvularia* is another important genus in this family. There are many reports of *Curvularia* species from Poaceae, including rice in the world [44,45,58,63,68,103,104]. We report three species of *Curvularia* including *C. geniculata* and *C. plantarum* as saprobes, and *C. chiangmaiensis* associated with leaf spots. Marin-Felix et al. [60] introduced five new species of *Curvularia* from different monocotyledon plants in Chiang Mai, but they did not report any strains from rice. Ferdinandez et al. [44,45] showed a high diversity of *Curvularia* species in cereal crops including rice in Sri Lanka. *Curvularia plantarum*, was first reported from *Saccharum officinarum* in China [68], and was also isolated from rice as a new host record in Sri Lanka [45]. In the current study, we recorded *C. plantarum* as the first geographical report from Thailand.

*Phaeosphaeriaceae* is another important and large family within Pleosporales, playing a crucial role as plant pathogens and causing infections in significant agricultural crops [105,106]. In addition, numerous species are saprophytes on monocotyledons, particularly on poaceous hosts. Some species may exist as saprobes on herbaceous dicotyledonous hosts, while others can act as endophytes [18,70]. While *Phaeosphaeria musae* has been documented on various plant families including Arecaceae, Asparagaceae, Marantaceae, and Musaceae [71,107,108,109], its presence in rice was not previously reported. Moreover, phylogenetic analyses revealed that two sexual strains and one asexual strain isolated in this study clustered together with *P. musae*, which was previously known only from its sexual morph. Hence, we report the asexual form of this species for the first time. Another genus within *Phaeosphaeriaceae*, *Setophoma*, was initially described by de Gruyter et al. [80]. Phookamsak et al. [110] illustrated the sexual form of *Setophoma sacchari* and *S. poaceicola* [74] for the first time from Thailand. In the current study, two strains of *S. poaceicola* demonstrated its asexual morph for the first time. *Setophoma* species have been reported from numerous host plants as well as members of the family Poaceae (e.g., sugarcane, corn, grasses) [74,111,112,113,114,115,116]. However, an association between *Setophoma* and rice had not been documented before. *Ophiosphaerella* species are globally reported as pathogens or saprobes on Poaceae, Cyperaceae, Asparagaceae, and Zingiberaceae [23,71,76,77,117,118]. Wetzel et al. [119], Hutchens et al. [120], and Tomioka et al. [121] have recorded a variety of *Ophiosphaerella* species isolated from grasses and cereal crops. The present study reports the first association between *Ophiosphaerella* and rice.

*Pyrenochaetopsidaceae* contains three genera *Neopyrenochaetopsis*, *Pyrenochaetopsis*, and *Xenopyrenochaetopsis.* Despite broad world distribution, this study reveals the first record of association between rice and *Pyrenochaetopsis*. Species of *Pyrenochaetopsis* are frequently characterized by their asexual morph but two studies have documented the sexual morph of *Pyrenochaetopsis* [20,43]. In the present study, the sexual morphs of *P. sinensis* were isolated as a new host record, and *P. oryzicola* was described as a new species.

*Didymellaceae* encompasses cosmopolitan species with a broad distribution. More than 50% of its members are plant pathogens causing great losses to a wide range of economic crops [83,122]. *Epicoccum*, a significant genus within this family, can be found in the air, soil, human toenails, diverse plant material, and aquatic environments [86,123,124,125]. Plant-associated species have different lifestyles including endophytes [126]. *Epicoccum nigrum* and *E. sorghinum* have been reported on rice causing brown blotches on glumes and leaf spot disease, respectively [127,128,129]. We isolated three strains of *E. catenisporum* and one of *E. latusicollum*. *Epicoccum catenisporum* was isolated from leaf spots of rice in 1978 and deposited as an ex-holotype living culture, which was later discovered as a novel species by Valenzuela-Lopez et al. [78]. *Epicoccum latusicollum* was isolated as a pathogen associated with rice sheath rot in Ethiopia [130]. However, scant and insufficient information was provided on both morphology and phylogeny to verify the species identification. Herein, *E. latusicollum* is reported as a new geographical record from Thailand, and identified as saprobe on rice.

Species of *Didymosphaeriaceae* are often saprobic [21]. Two genera, *Pseudopithomyces* and *Paraphaeosphaeria*, were isolated in our study. *Pseudopithomyces chartarum* is recognized as the causal agent of leaf spot diseases affecting a variety of plants, including medicinal plants, grasses, cereals, and legumes [131,132,133,134,135,136]. Instances of *P. chartarum* as a pathogen from rice have been documented in India, New Zealand, and Brazil [92,137,138]. *Paraphaeosphaeria* species have been isolated from different hosts and substrates such as tea, sugarcane, coconut, rotten wood, dead leaves, soil, and fresh water [89,139,140]. Herein, a new species of *Paraphaeosphaeria* was isolated from rice.

Studying fungal species diversity on rice will help to improve agricultural practices, protect ecosystems, enhance economic outcomes, and ensure global food security. Establishing a comprehensive understanding of current fungal diversity provides a baseline for monitoring changes over time.

## Figures and Tables

**Table 1:** Details of genes/loci and PCR primers used in this research.

Loci	Primer Pairs (Forward/Reverse)	Reference
LSU	LROR 5′–ACCCGCTGAACTTAAGC–3′ LR5 5′–ATCCTGAGGGAAACTTC–3′	[34]
ITS	ITS5 5′–GGAAGTAAAAGTCGTAACAAGG–3′ ITS4 5′–TCCTCCGCTTATTGATATGC–3′	[35]
SSU	NS1 5′–GTAGTCATATGCTTGTCTC–3′ NS4 5′–CTTCCGTCAATTCCTTTAAG–3′	[35]
*tef*1	983F 5′–GCY CCY GGH CAY CGT GAY TTY AT–3′ 2218R 5′–AT GAC ACC RAC RGC RAC RGT YTG–3′	[36]
*rpb*2	fRPB2–5f 5′–GGG GWG AYC AGA AGA AGG C–3′ fRPB2–7cR 5′–GGG GWG AYC AGA AGA AGG C–3′	[37]
*tub*2	BT2a 5′–GGTAACCAAATCGGTGCTGCTTTC–3′ BT2b 5′–ACCCTCAGTGTAGTGACCCTTGGC–3′	[38]
	T1 5′–AAC ATG CGT GAG ATT GTA AGT–3′ T2 5′–TAG TGA CCC TTG GCC CAG TTG–3′	[34]
*gapdh*	Gpd1 5′–ATT GGC CGC ATC GTC TTC–3′ Gpd2 5′–CCC ACT CGT TGT CGT ACC–3′	[39]

**Table 2:** Details of the species obtained in this study.

Species	Strain Number	Host Part	Location	Life-Style	Cultivar
*Bipolaris chiangraiensis*	MFLUCC 24-0016	Panicle	Tha Sut sub-district, Mueang Chiang Rai District	Saprobic	RD6
*B. oryzae*	MFLUCC 24-0010	Panicle	Mueang Chiang Rai District	Saprobic	Unknown
	MFLUCC 24-0011	Panicle	Mueang Chiang Rai District	Saprobic	Unknown
	MFLUCC 24-0012	Panicle	Phan District	Saprobic	RD6
	MFLUCC 24-0013	Panicle	Phan District	Saprobic	RD15
	MFLUCC 24-0014	Stem	Huai Sak sub-district, Mueang Chiang Rai district	Saprobic	RD15
	MFLUCC 24-0015	Panicle	Tha Sut sub-district, Mueang Chiang Rai District	Saprobic	RD6
	MFLUCC 24-0017	Panicle	Tha Sut sub-district, Mueang Chiang Rai District	Saprobic	RD6
	MFLUCC 24-0018	Leaf	Si Mueang Chum sub-district, Mae Sai District	Endophytic	RD-MAEJO2
*Curvularia chiangmaiensis*	MFLUCC 24-0024	Leaf	Mueang Chiang Rai District	Associated with leaf spots	Pathum Thani 60
*C. geniculata*	MFLUCC 24-0022	Panicle	Wiang Chai sub-district, Wiang Chai District	Saprobic	RD6
*C. plantarum*	MFLUCC 24-0021 MFLUCC 24-0023	Panicle Stem	Phan District Phan District	Saprobic Saprobic	RD15 KDML105
*Epicoccum catenisporum*	MFLUCC 24-0025 MFLUCC 24-0027 MFLUCC 24-0028	Seedling Stem Seedling	Mae Chan District Phan District Thung Ko sub-district, Wiang Chiang Rung District	Associated with leaf spots Saprobic Associated with leaf spots	Pathum Thani 60 KDML105 RD6
*E. latusicollum*	MFLUCC 24-0026	Leaf	Si kham sub-district, Mae Chan District	Saprobic	Pathum Thani 60
*Ophiosphaerella agrostidis*	MFLUCC 24-0029	Stem	Huai Sak sub-district, Mueang Chiang Rai District	Saprobic	RD15
*O. oryzae*	MFLUCC 24-0030	Stem	Huai Sak sub-district, Mueang Chiang Rai District	Saprobic	RD15
*Paraphaeosphaeria oryzae*	MFLUCC 24-0032	Leaf	Thung Ko sub-district, Wiang Chiang Rung District	Associated with leaf spots	RD6
*Phaeosphaeria musae*	MFLUCC 24-0044	Panicle	Mueang Chiang Rai District	Saprobic	Unknown
	MFLUCC 24-0045	Panicle	Mueang Chiang Rai District	Saprobic	Unknown
	MFLUCC 24-0046	Panicle	Si kham sub-district, Mae Chan District	Saprobic	Pathum Thani 60
*Pseudopithomyces chartarum*	MFLUCC 24-0047	Panicle	Phan District	Saprobic	RD15
	MFLUCC 24-0048	Leaf	Thung Ko sub-district, Wiang Chiang Rung District	Associated with leaf spots	RD6
*Pyrenochaetopsis indica*	MFLUCC 24-0036	Panicle	Phan District	Saprobic	RD6
	MFLUCC 24-0037	Stem	Huai Sak sub-district, Mueang Chiang Rai District	Saprobic	RD15
*P. oryzicola*	MFLUCC 24-0038	Panicle	Phan district	Saprobic	RD15
	MFLUCC 24-0039	Stem	Huai Sak sub-district, Mueang Chiang Rai District	Saprobic	RD15
	MFLUCC 24-0040	Stem	Huai Sak sub-district, Mueang Chiang Rai District	Saprobic	RD15
	MFLUCC 24-0041	Panicle	Tha Sut sub-district, Mueang Chiang Rai District	Saprobic	RD6
	MFLUCC 24-0042	Panicle	Tha Sut sub-district, Mueang Chiang Rai District	Saprobic	RD6
*P. sinensis*	MFLUCC 24-0043	Leaf	Si Mueang Chum sub-district, Mae Sai District	Associated with leaf spots	RD-MAEJO2
*Remotididymella capsici*	MFLUCC 24-0031	Seedling	Wiang Chai sub-district, Wiang Chai District	Endophytic	MAEJO
*Setophoma oryzicola*	MFLUCC 24-0035	Stem	Phan district	Saprobic	KDML105
*S. poaceicola*	MFLUCC 24-0033	Panicle	Wiang Chai sub-district, Wiang Chai District	Saprobic	RD6
	MFLUCC 24-0034	Stem	Phan District	Saprobic	KDML105

## Data Availability

All sequence data are available in NCBI GenBank following the accession numbers in the manuscript.

## Supplementary Materials

The following supporting information can be downloaded at: https://www.mdpi.com/article/10.3390/jof10110763/s1, Figure S1: *Bipolaris oryzae* (MFLU 24-0089); Figure S2: *Curvularia chiangmaiensis* (MFLU 24-0196); Figure S3: *Curvularia geniculata* (MFLU 24-0092); Figure S4: *Curvularia plantarum* (MFLU 24-0091, new geographical record); Figure S5: *Ophiosphaerella agrostidis* (MFLU 24-0094, new host record); Figure S6: *Pyrenochaetopsis indica* (MFLU 24-0197, new host record); Figure S7: *Epicoccum catenisporum* (MFLU 24-0093, new geographical record); Figure S8: *Epicoccum latusicollum* (MFLU 24-0317, new host record); Figure S9: *Remotididymella capsici* (MFLUCC 24-0031, new host and geographical record); Figure S10: *Pseudopithomyces chartarum* (MFLU 24-0101).
