# Supplementary material for: Diversity of Pleosporalean Fungi Isolated from Rice (Oryza sativa L.) in Northern Thailand and Descriptions of Five New Species
Source: J Fungi (Basel). 2024 Nov 2;10(11):763. doi: 10.3390/jof10110763 (PMC11595767; doi:10.3390/jof10110763)
Supplement: Supplementary file 1 [file jof-10-00763-s001.zip › jof-3290032-supplementary.pdf]

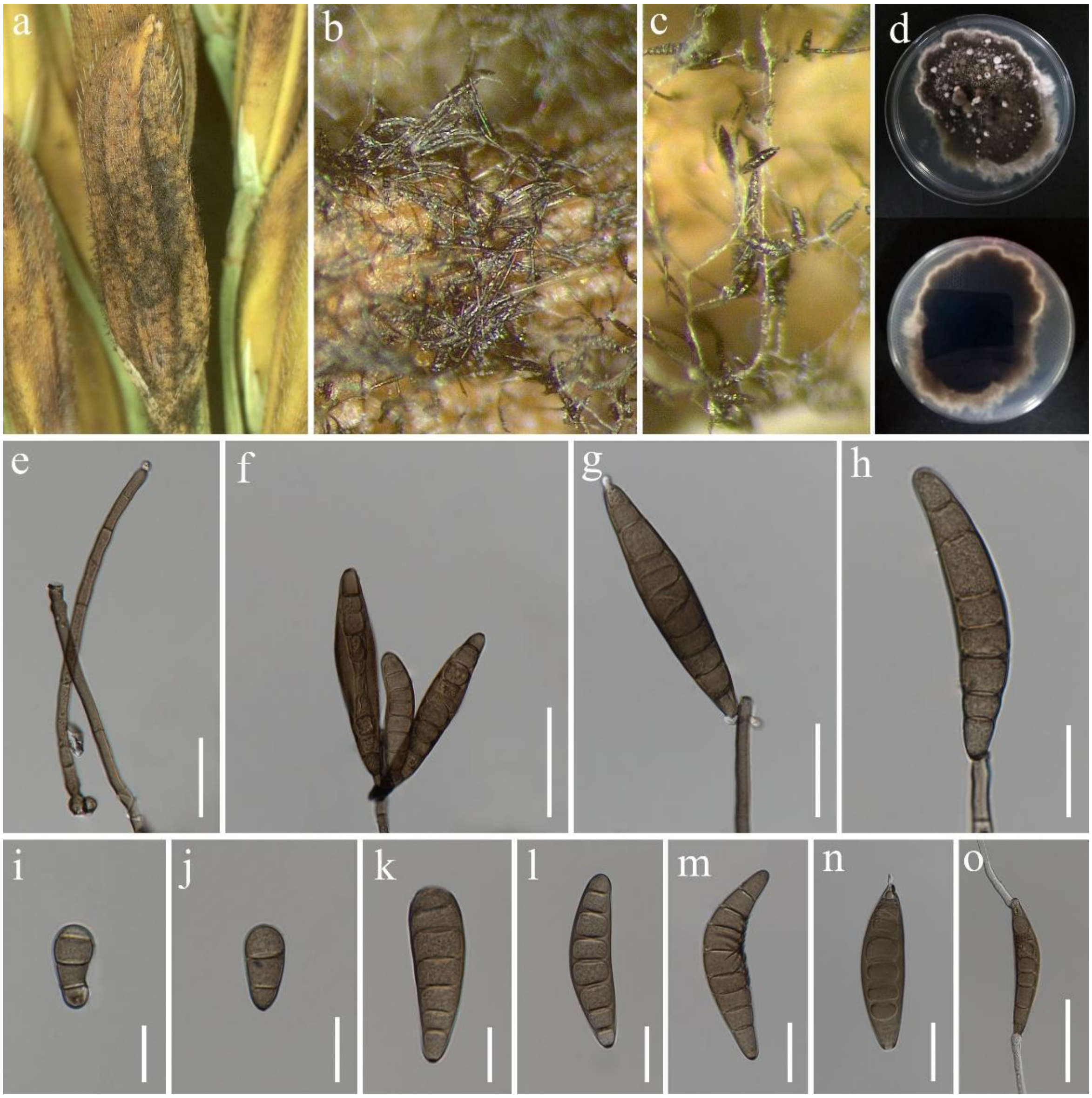

Figure S1. *Bipolaris oryzae* (MFLU 24-0089). (**a–c**) Conidiophores and conidia on panicle of *Oryza sativa*; (**d**) Top and reverse of colony on PDA; (**e**) Conidiophores; (**f–h**) Conidiogenous cells and conidia; (**i–n**) Conidia from immature to mature; (**o**) Germinating conidium. Scale bars: (**e–h**) = 50 μm; (**i–o**) = 20 μm.

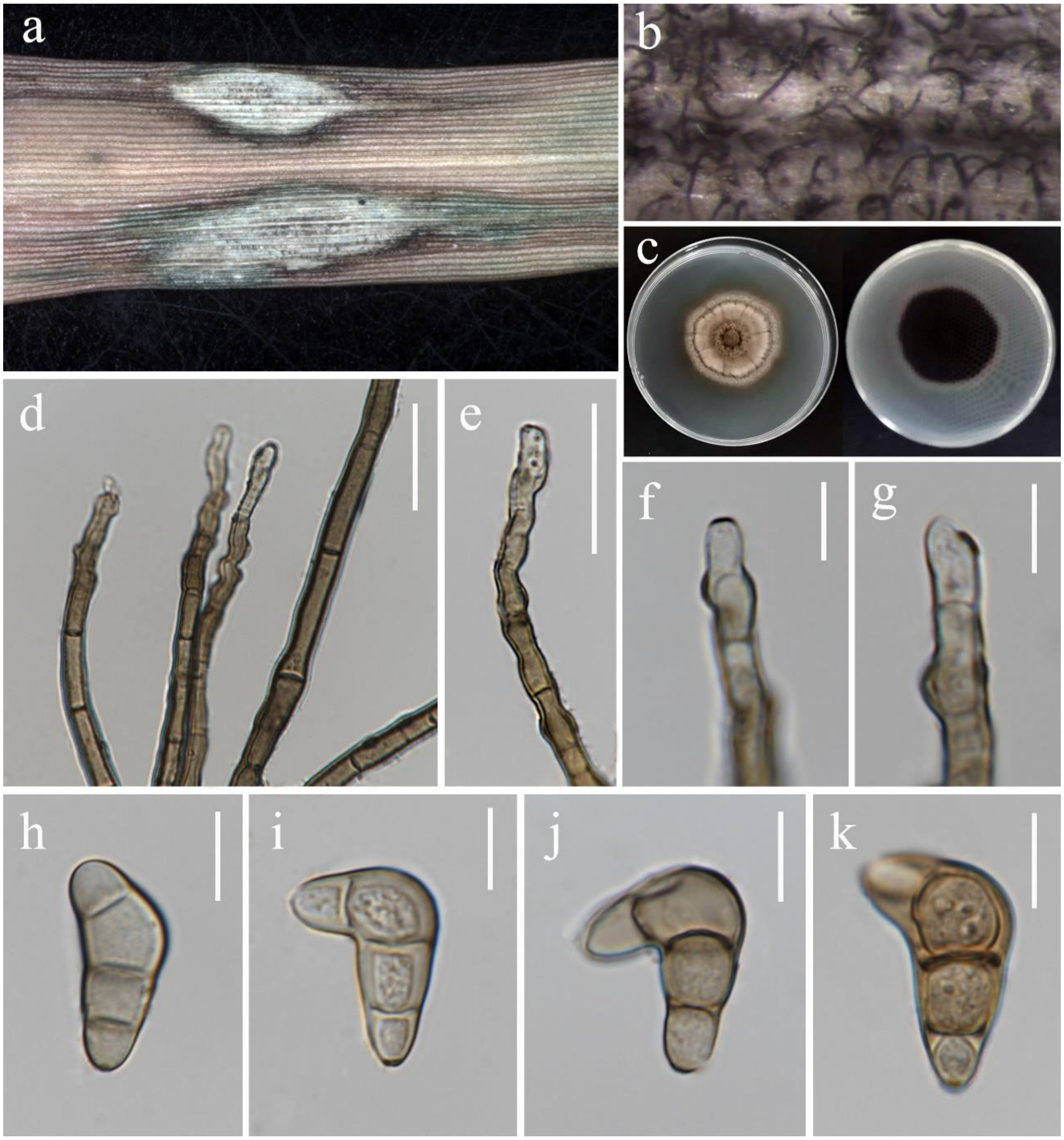

Figure S2. *Curvularia chiangmaiensis* (MFLU 24-0196). (**a**) Leaf spots on *Oryza sativa*; (**b**) Conidiophores and conidia on the host; (**c**) Top and reverse of colony on PDA; (**d–g**) Conidiophores and conidiogenous cells; (**h–k**) Conidia. Scale bars: (**d–e**) = 20 μm; (**f–k**) = 10 μm.

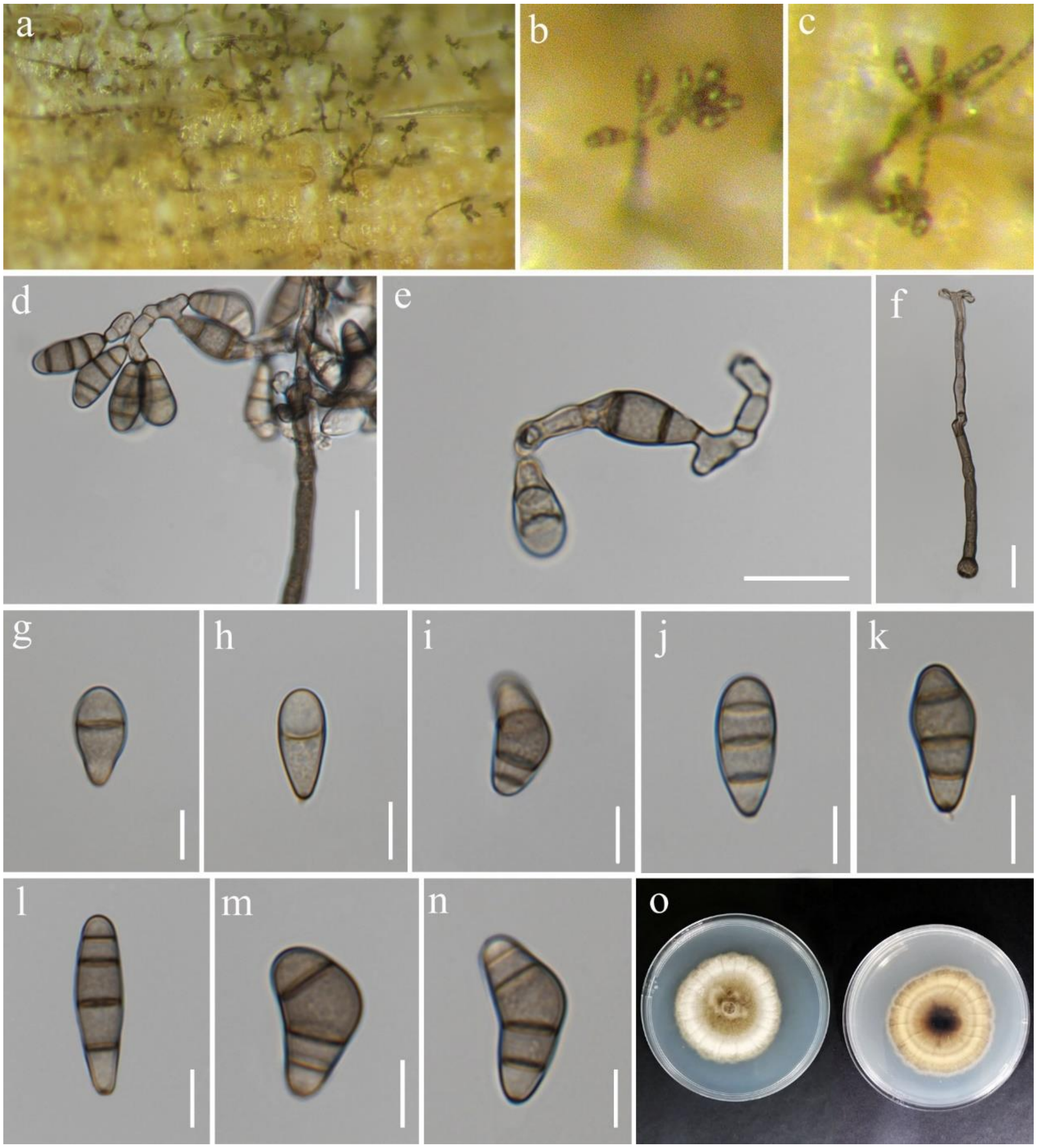

Figure S3. *Curvularia geniculata* (MFLU 24-0092). (**a–c**) Conidiophores and conidia on the panicle of *Oryza sativa;* (**d–e**) Conidiogenous cells and conidia. (**f**) Conidiophore. (**g–n**) Conidia. (**o**) Top and reverse of colony on PDA. Scale bars: (**d–f**) = 20 μm; (**g–n**) = 10 μm.

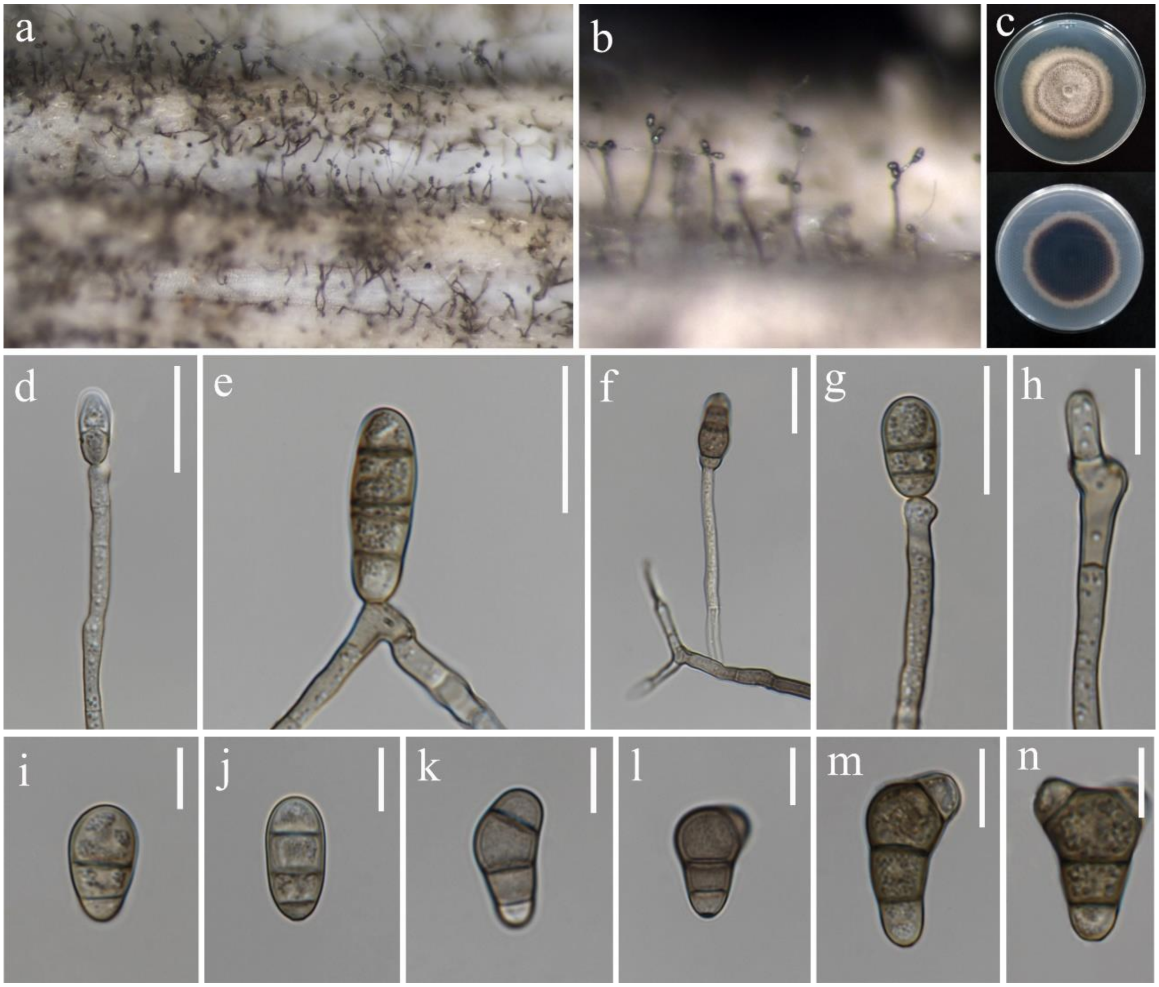

Figure S4. *Curvularia plantarum* (MFLU 24-0091, new geographical record). (**a–b**) Conidiophores and conidia on the stem of *Oryza sativa*; (**c**) Top and reverse of colony on PDA; (**d–g**) Conidiogenous cells and conidia; (**h**) Conidiophore; (**i–n**) Conidia. Scale bars: (**d–g**) = 20 μm; (**h–n**) = 10 μm.

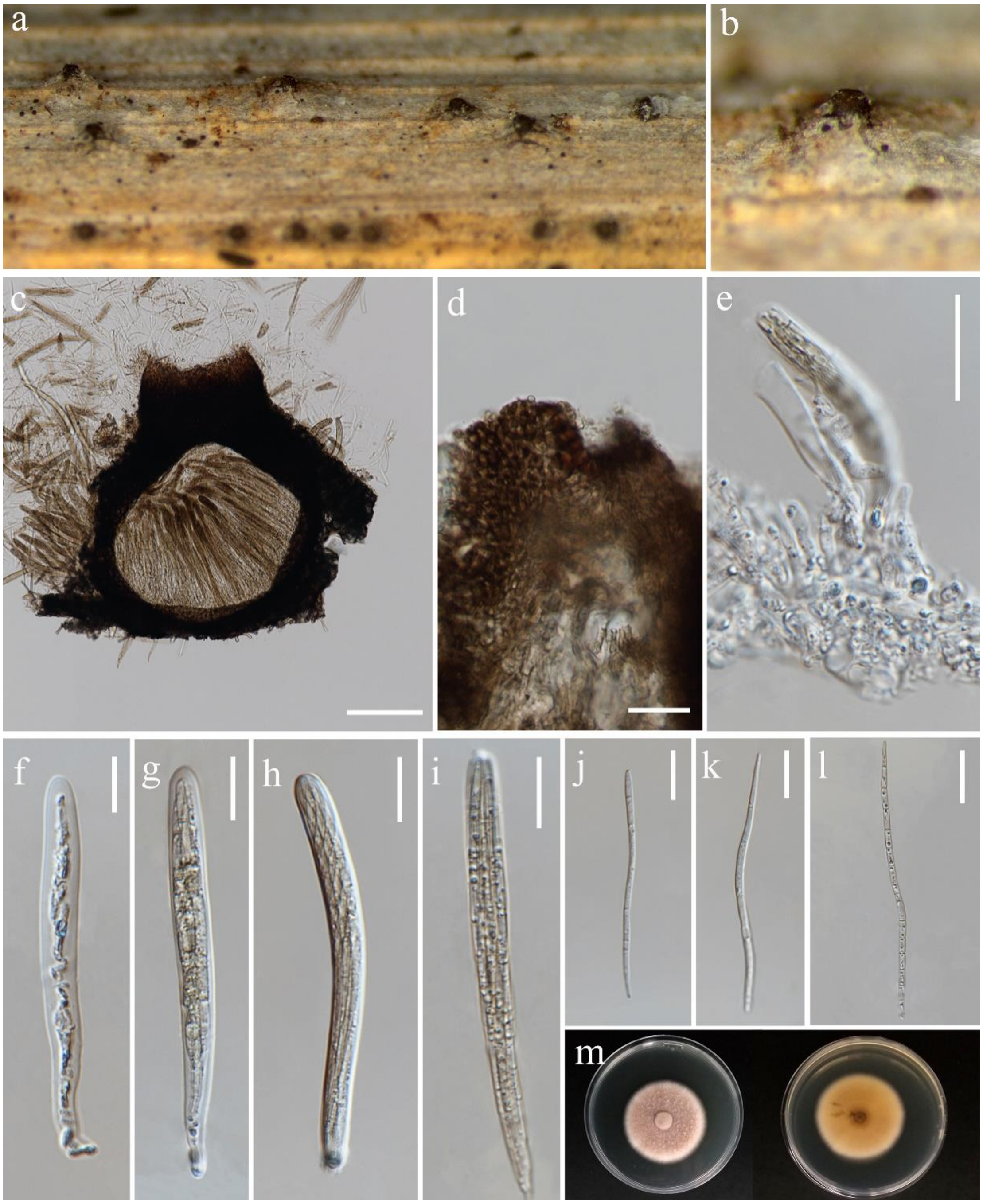

Figure S5. *Ophiosphaerella agrostidis* (MFLU 24-0094, new host record). (**a–b**) Appearance of ascomata on the stem of *Oryza sativa*; (**c**) Section through ascoma; (**d**) Ostiole; (**e**) Ascogenous cells; (**f–i**) Asci; (**j–l**) Ascospores; (**m**) Top and reverse of colony on PDA. Scale bars: (**c**) = 100 μm; (**d–l**) = 20 μm.

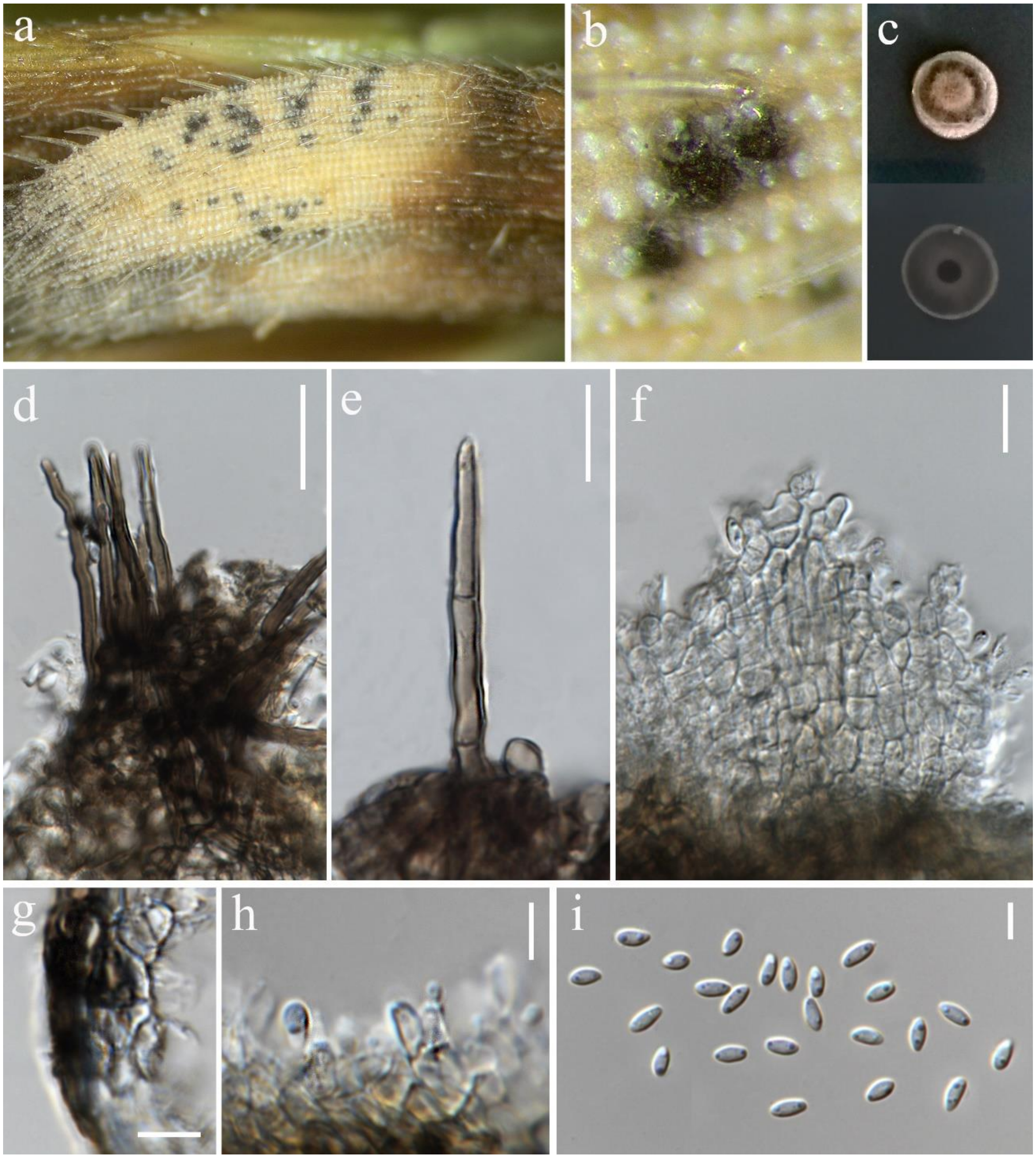

Figure S6. *Pyrenochaetopsis indica* (MFLU 24-0197, new host record). (**a**–**b**) Appearance of conidiomata on the panicle of *Oryza sativa*; (**c**) Top and reverse of the colony on PDA; (**d**–**e**) setae; (**f**–**g**) Pycnidial wall; (**h**) Conidiogenous cells; (**i**) Conidia. Scale bars: (**d**) = 20 µm; (**e**–**f**) = 10 µm; (**g**–**i**) = 5 µm.

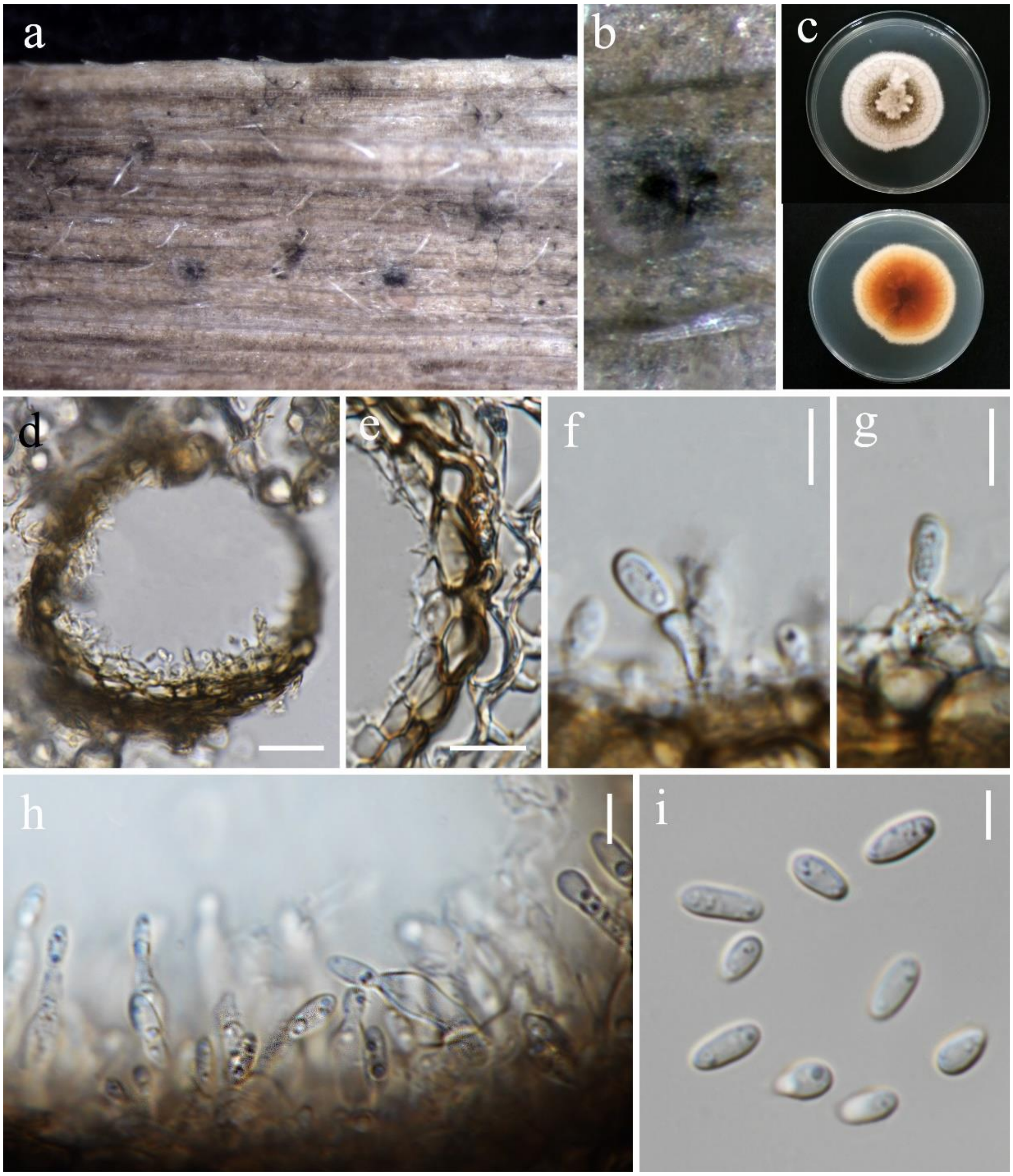

Figure S7. *Epicoccum catenisporum* (MFLU 24-0093, new geographical record). (**a–b**) Appearance of conidiomata on the leaf of seedling of *Oryza sativa*; (**c**) Top and reverse of colony on PDA; (**d**) Section through pycnidium; (**e**) Pycnidial wall; (**f–h**) Conidiogenous cells; (**i**) Conidia. Scale bars: (**d**) = 20 μm; (**e**) = 10 μm; (**f–i**) = 5 μm.

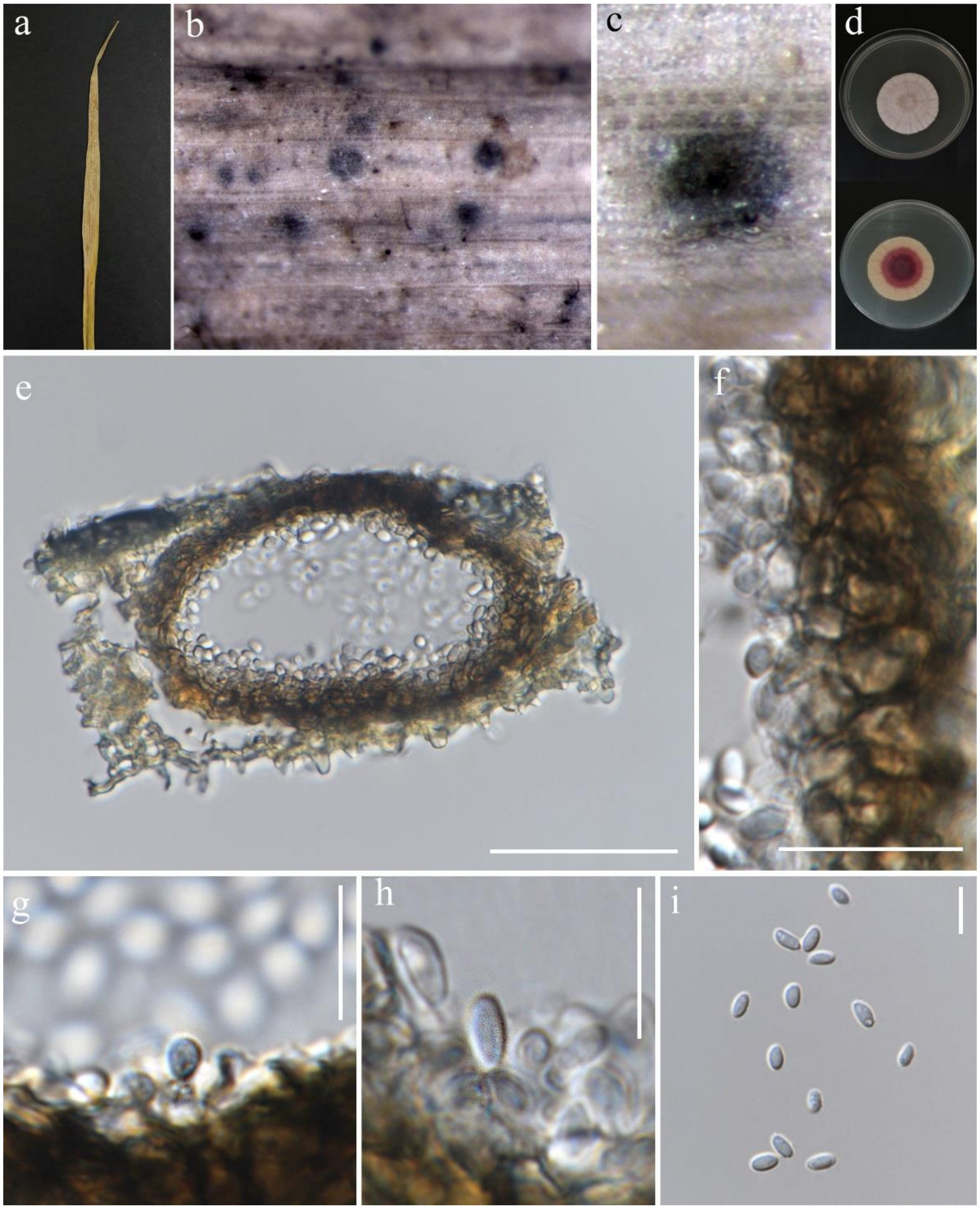

Figure S8. *Epicoccum latusicollum* (MFLU 24-0317, new host record). (**a**) Appearance of substrate; (**b–c**) Appearance of conidiomata on dead leaf of *Oryza sativa*; (**d**) Top and reverse of colony on PDA; (**e**) Section through pycnidium; (**f**) Pycnidial wall; (**g–h**) Conidiogenous cells; (**i**) Conidia. Scale bars: (**e**) = 50 μm; (**f–i**) = 10 μm.

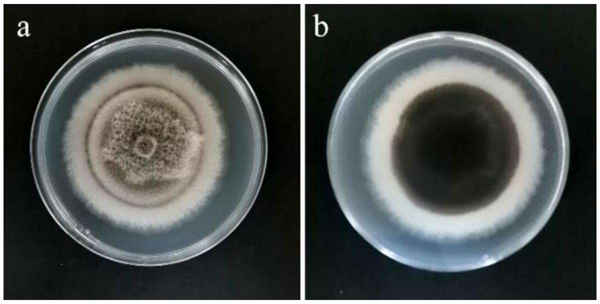

Figure S9. *Remotididymella capsici* (MFLUCC 24-0031, new host and geographical record). (**a**–**b**) Top and reverse of colony on PDA.

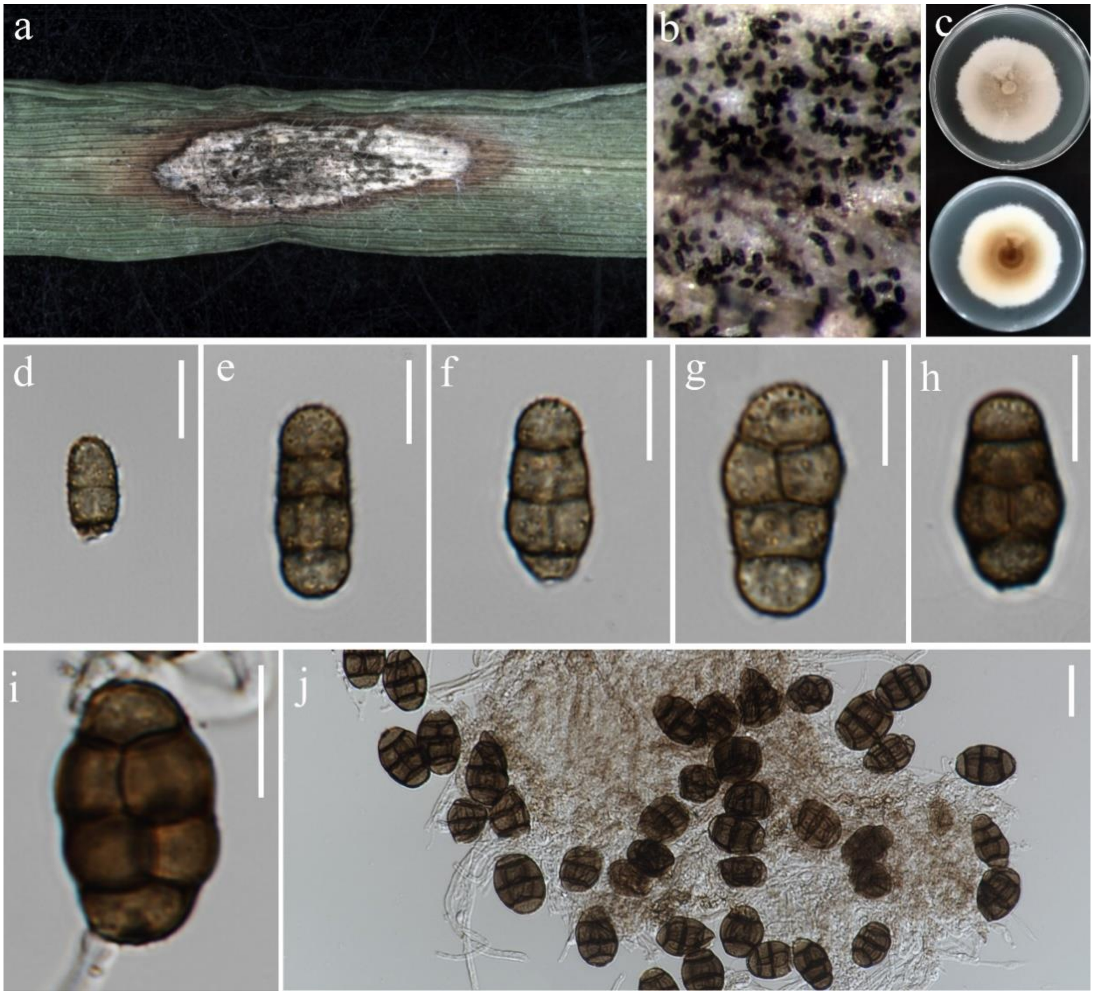

Figure S10. *Pseudopithomyces chartarum* (MFLU 24-0101). (**a**–**b**) Appearance of spores on the leaf spot of *Oryza sativa*; (**c**) Top and reverse of colony on PDA; (**d**–**i**) Conidia; (**j**) Conidiophores and conidia. Scale bars: (**d**–**i**) = 10 μm; (**j**) = 20 μm.
